# Who sells to whom in the suburbs? Home price inflation and the dynamics of sellers and buyers in the metropolitan region of Paris, 1996–2012

**DOI:** 10.1371/journal.pone.0213169

**Published:** 2019-03-21

**Authors:** Renaud Le Goix, Timothée Giraud, Robin Cura, Thibault Le Corre, Julien Migozzi

**Affiliations:** 1 Université Paris Diderot USPC / UMR Géographie-cités 8504 CNRS, Paris, France; 2 Université Paris Diderot USPC / UMS RIATE CNRS, Paris, France; 3 Université Paris 1 Panthéon Sorbonne / UMR Géographie-cités 8504 CNRS, Paris, France; 4 Ecole Normale Supérieure de Paris / UMR PACTE, Grenoble, France & UMR Géographie-cités 8504, Paris, France; University of Lausanne, SWITZERLAND

## Abstract

Price inflation has outbalanced the income of residents and buyers in major post-industrial city-regions, and real estate has become an important driver of these inequalities. In a context of a resilient inflation of home values during the last two decades in the greater Paris Region, it is critical to examine housing price dynamics to get a better understanding of socioeconomic segregation. This paper aims at presenting spatial analysis of the dynamics of segregation pertaining to inflation, analyzing price and sellers and buyers data. Using interpolation techniques and multivariate analysis, the paper presents a spatial analysis of property-level data from the Paris Chamber of Notaries (1996-2012) in a GIS (159,000 transactions in suburban areas, single family homes only). Multivariate analysis capture price change and local trajectories of occupational status, i.e. changes in balance between inward and outward flows of sellers and buyers. We adopt a method that fits the fragmented spatial patterns of suburbanization. To do so, we remove the spatial bias by means of a regular 1-km spatial grid, interpolating the variables within it, using a time-distance matrix. The main results are threefold. We document the spatial patterns of professionalization (a rise of executives, intermediate occupation and employees) to describe the main trends of inward mobility in property ownership in suburbs, offsetting the outward mobility of retired persons. Second, neighborhood trajectories are related the diverging patterns of appreciation, between local contexts of accumulation with a growth of residential prices, and suburbs with declining trends. The maturity of suburbanization yields a diversified structure of segregation between the social groups, that do not simply oppose executives vs. blue collar suburbs. A follow-up research agenda is finally outlined.

## Introduction

In France, continuous inflation of residential real estate for the last 20 years and after the financial crisis has remained a paradox: while the evolution of both price to income ratio (i.e. affordability index) and rent to price ratio [1] should discourage homebuyers and investors in French metropolitan areas, housing markets have remained active and the price trend did not reverse [2, 3], a situation that Timbeau [4] analyzed as a “resilient bubble”. This issue forms the basis of our research. To analyze this paradox, we elaborate on Piketty’s statement (2013), that real estate assets have become a main driver of socio-spatial inequalities among households, the flow of investment by households being instrumental in the dynamics of asset capitalization and segregation. Housing finance and credit have expanded dramatically, and explain this flow of investment [5–7], yielding a price inflation regime.

This paper seeks to provide an empirically grounded, neighborhood based, visualization and analysis of housing market dynamics, that elaborates on how price inflation has increased socioeconomic segregation. We therefore assume that real estate assets and prices have become the main driver of socio-spatial inequalities among households. Lower income households have gained access to property ownership through the expansion of credit, affordability and subsidized access to ownership. But we also assume that the price inflation regime has been produced by an unprecedented flow of credit and transmission of assets (i.e. intergenerational transfers) fueling price inflation and inequalities.

Price inflation has rendered the structure of affordability more inequitable, thereby exacerbating socio-spatial exclusion and segregation. This issue requires a careful examination of the representation and conditions of local markets. To do so, we examine the single family home market in the suburbs of Paris metropolitan region, structured as a 60% owner occupied market. Mapping and spatially analyzing residential markets matter, because such representations structure knowledge and decisions by market actors and homebuyers. For instance, households secure their own trajectories, capitalizing on real-estate assets and/or engaging with risk and financial vulnerability [8]. In French metropolitan areas, the spatial patterns of price inflation, however, confuse the signals property buyers generally anticipate from the market. While inflation seems ubiquitous, it has increased the spatial homogeneity between neighborhoods (a strong negative correlation between initial price level and its increase leads to a trend of spatial homogenization), but it has paradoxically also been said to have strengthened the unequal spatial structure of price and the hierarchy of neighborhoods [9–12].

We assume, to a certain extent, that the theoretical framework linking property price and socioeconomic inequalities is not fully stabilized. Indeed, there are two relatively independent extensive bodies of academic literature. On the one side, much has been written about the financialization of housing, that will be discussed in the literature review (section 1). On the other, much has been done to explain and model local house price variations (for instance, but not exclusively, econometrics of housing prices, discussed in section 2). We aim at combining these approaches in an integrated analysis of the dynamic of property prices and the dynamics of inequalities and segregation, to better address local patterns and dynamics.

- This requires offsetting the limits of the commonly stylized relationships between segregation, inequalities and housing prices. Indeed, most of the literature about economic inequalities is based on the fact that asset inequalities between households depend on housing values, and most of the standard approaches of housing markets assume that large parts of the differences between real estate values depend upon social and urban parameters, *e.g*. the socioeconomic composition of the neighborhood. To put it simply, property values are in one approach an independent variable, and in another one a dependent variable.
- The research therefore aims at building a more systemic understanding of the relationships between housing prices and inequalities [13]. A spatio-temporal approach of housing affordability and social structure of ownership is necessary to avoid the risk of decontextualized views of inequalities and housing dynamics. We do so by bridging datasets describing individual transactions from the Chamber of the Notaries (base BIEN) and spatial grids, to unfold a mapping effort that takes into account the variegated and fragmented spatial patterns of suburbanization.

In this context, the novelty of this paper is to deal with (1) social change with individual data, in a property owners market, not relying on decennial census data but rather on annual flows of sellers and buyers. Such variables describing individuals in transaction data are rare but highly informative. Compared to decennial census data and sample population public data, doing so allows us to get a better understanding of the market dynamics occurring between censuses, and for instance to capture the effects of the 2007-2008 global financial crisis (GFC), and its recovery. We analyze a geospatial dataset provided by the Paris Chamber of the Notaries, describing individual transactions between 1996 and 2012. As a corollary, we also aim at (2) adopting an adequate methodology to handle discrete individual and spatially indexed socioeconomic data to draw longitudinal analysis and compare data over time. We remove the spatial bias by establishing a regular spatial grid, interpolating the variables within it.

The paper is structured as follows: The first section covers the background and literature review, insisting on the following paradox: an increased income to value gap did not impact resilient market dynamics, in a context of increased financialization and asset-based welfare transition. We frame the Paris case study in the literature as a model of capitalist urban production. A subsequent section sets the empirical grounds and describes our methodological approach, designed to address the challenges of real-estate data analysis within suburban / exurban areas. We finally describe and discuss the findings of this research, a spatial analysis of neighborhood change (upward/downward) produced by market dynamics: price, transactions, seller-buyers socio-occupational categories. Elaborating on the results of this study, the conclusion outlines a research agenda connecting inequalities between households, affordability of housing and wealth accumulation in owner-occupied properties.

## Research background: Analyzing the price regime and socio-economic dynamics of suburban markets in the Paris region

Price and income have been correlated for a long period of time, a strong linkage that Friggit compared to a “tunnel” of price [1]. The gap between property prices and household income has widened since the early 2000s: affordability is now at its lowest historical level since 1965, with property values increasing over 70% since 2000 [1]. For a time, households benefited from lower interest rates and longer loan maturities to offset the effects of price inflation and to maintain purchasing power. The following paradox has consequently emerged in the post-financial crisis era: while the evolution of both price-to-income ratio (i.e. affordability index) and rent-to-price ratio should discourage homebuyers and investors in metropolitan areas (in France, and also in other national contexts), housing markets have remained active and the price trend did not reverse. Most public policies have actively reinforced ownership as a standard lifestyle. The homeownership rate has increased from 35% in 1954, up to 57% [14], a movement analyzed as the outcome of a patrimonial middle class benefiting from price growth, government incentives for credit, and securing life trajectories [8, 15].

### Volatile inflation and market regimes

Since the early 1990s, housing finance has increased dramatically in the Global North, yielding a volatile inflation of price: residential mortgage outstanding debt reached historical levels in 2006: 35% (France), 50%(Sweden, Spain, Germany), 80% (US) and 100% (Netherlands, Denmark) of the GDP, according to Schwartz and Seabrooke [16]. Since the early 2000s, a gap between household income and property price has therefore widened. In the US, Australia, Britain, Ireland, Canada, the average house price-to-income ratio of 3:1 in 1996 reached values between 5:1 and 4:1 in 2007 [16]. A study conducted by Aalbers in 2016 [17] in 17 countries (14 in Europe, and USA, Canada, Japan) showed that the homeownership rates ranged between 50 to 80%, while change in 1985-2010 price-to-income ratio increased from + 13% up to +28% in France, 44% in the UK; some exceptions being Germany and Japan.

In cities with dynamic residential markets, property price inflation is generally explained by a shortage of supply in face of an excessive demand. To avoid a crisis, the proposed solutions are supply-side policies that seek to redress the market balance by expanding housing provision. Yet such measures are likely to stimulate inflation [18]. The reason is that housing shortage in local markets is not independent from other factors that are often analyzed at macro-economic levels as (1) market arrangements, driven by the generalization of mortgage to lower income households in post-industrial economies [5–7]; (2) aging of homeowners, and induced intergenerational equity gaps between retirees (75% are owners in France) and the rest of the population [19]; (3) global financial strategies that contribute to reduce the supply of affordable housing in major metropolitan areas [20, 21]; (4) regulation and state-led incentives supporting both lower-income homeownership [22] and rental investment [23]; (5) ordinary financialization, *i.e*. household’s wealth dependency to market fluctuations and necessity for households to behave as if they were assets managers, because of the role of housing wealth to secure life trajectories [24, 25].

### Homeownership and vulnerability of households

These are the reasons why Aalbers [17] contends that among the variegated forms of contemporary capitalism and real-world political economy, housing, capital accumulation, debt and price are central issues, yet peripheral to academic political economy and geography. Indeed authors theoretically describe a path dependency-shift in almost every nation-state influenced by global World Bank policies [26], after the 1993 report *Housing: Enabling Markets to Work summarized assessment, best practices and recommendations* on how important the housing sector could be for the economy, including guidelines and suitable policies for governments. In a political economy perspective, Fernandez and Aalbers [27] argue that despite significant differences, a gradual shift across national models has occurred. Scholarly works describe housing asset-based welfare, a rising ideology of homeownership across modern-industrialized societies [28], characterized by liberal-economic ideologies and market mechanisms driven by policy-measures, socio-ideological frameworks [29] and finance-led accumulation regimes [30]. More specifically, theories link the high proportion of capital investment that housing represents for households and the ways welfare states are organized and reformed [31], the pursuit of owner-occupancy promotion being viewed as a superior form of tenure, while privatizing social housing [32]. Trajectories are then highly dependent on national frameworks, and the state enables households to act as investors, engaging through markets with prospects of future gains, while exposed to greater risks (volatility of price, loss of property values, risks of bankruptcy and foreclosures, etc.), raising questions of individual and systemic risks, and therefore vulnerabilities of households [25]. This statement is also true in the push for a homeowners’ society in France [15].

### Characterizing suburban production regime in France

Many changes have affected the suburban housing expansion regime since the 1980s, that have not been thoroughly addressed and characterized in France, compared to the existing literature in Spain [33–35], in the US [5, 7, 36], in South-East Asia [18, 37], and the expansion of low-income housing financing in the Global South [38–40]. Homeownership in France, implemented as a means to secure the life trajectory of households, can be also analyzed as a way to reform welfare and the ordinary financialization of social/retirement insurance, which is a social process inducing inequalities and uncertainty [14, 15]. The making of the new institutional regime is not only national but also local. Housing policies, relationships between stakeholders, financial institutions, planners, builders, and regulators are embedded into local/regional local contexts that shape property market dynamics [41]. This contributes to the reinforcement of a new institutional regime where residential markets are seen as growth drivers with inflationary effects [3]. Public and private actors have converging interests in housing market expansion (fiscal resources, economic opportunities) and produce a specific representation of the housing demand [42, 43]. This attempt to solve what is seen as a “housing crisis” exclusively by expanding the housing provision has effects on households’ residential trajectories and asset strategies. This characterization of a new institutional regime leads to the question of whether it accentuates socio-spatial inequalities and segregation or not. Since the institutional regime relies on the mobilization of households, the financial effort (price-to-income ratio) appears as a key variable, as well as the socioeconomic origins of prospective buyers (i.e. sellers and buyers socio-occupational categories), the latter being the core variables analyzed in this paper.

### Theorizing suburbs in the metropolitan region of Paris as a property market case study

Not only the outcomes of changes in the suburban housing production regime have not been well characterized in France yet, but the theoretical significance of the Paris-region suburban case study, its historical and interpretative bias, are also to be discussed before we proceed further.

The city of Paris and its metropolitan region are often cited as a model, and as an exception, but are rarely actually included in comparative research. Compared to other A+ and A global cities [44] in which segregation and housing have been thoroughly analyzed and compared (i.e. New York, London, Tokyo, Chicago, Los Angeles, Hong-Kong and Shanghai), the immense body of literature discussing urban production, housing and segregation in Paris has been mostly published in the French language, and gives to Paris a relatively and regrettably exceptional standing in the field, starting with the setting up of the post-Second Empire Paris as a unique model of urban segregation, planning and public spaces. Often, Paris is compared to its European counterpart, London [45]. One hypothesis of this exceptionalism of the Paris case study may be related to the tremendous impact in urban studies of, for instance, Benjamin’s Baudelaire, Castells analysis of capitalism in the city [46], Lefebvre’s analysis downtown urban renewal [47], and Harvey’s *Paris, Capital of modernity* [48]. That is to say that urban production and segregation dynamics in Paris are therefore rarely compared to other cities, with the exception of Wacquant’s analysis of hyper-segregated inner suburbs of public housing, and his critics of comparison with the US afro-american ghettos [49].

Another explanation derives from of a certain idiosyncrasy of French language human geography itself, *i.e*. provincialism [50], in which the hyper-centralized Paris case study is first used to better comprehend the structure of inequalities at a national level: Paris-region segregation patterns are often compared to larger French metropolitan areas, so as to demonstrate how the capital region operates according to the global cities hypothesis of reinforced segregation patterns, compared to post-industrial socioeconomic patterns found in other French cities [51]. Although scholarly works in the French language thoroughly analyze segregation in Paris and its suburbs [52–56], segregation in the Paris metropolitan region, outside the limits of the inner city, is still scarcely documented in the English language: the recent *Socioeconomic segregation in European capital cities* excludes Paris [13], the second largest European capital. Recent work describing socioeconomic segregation and ethnic segregation also often elaborate on this relative exceptionalism of the Paris case study [57, 58]. Préteceille discusses this issue in a synthetic paper covering the dynamics of socioeconomic and ethno-racial segregation, social mixing and planning policies in Paris and its region [58]. To frame the research question, he insists on how the construction of debates on socioeconomic segregation in Paris has focused on explanations, with a mix of fascination and dramatization of segregation in the *banlieues*, with a US bias. Préteceille pushes for an approach that considers the processual and dynamic dimensions. His approach covers socioeconomic segregation (professionalization, mass unemployment and growing casualization of labor) and ethnic segregation by the means of census data, but his research does not cover how the transformation of the production of housing systematically contributes to inequalities, especially the contribution of suburban and mass homeownership to the process [58].

When segregation in France is however quantitatively analyzed in the English language, this is for instance to be compared with US socioeconomic segregation patterns, the theoretical focus being on cross-national variations on segregation, e.g. the size of the urban system and role of the welfare state in income redistribution [59]. Some focus has also been on categories of neighborhoods to compare, for instance revitalization in distressed areas, segregation and social mixing, comparing between Paris, and cities in North American and in the UK [60].

It is a corollary that the study of French and Parisian suburbs suffer from an American bias, suburban dynamics being easily matched to phenomena loosely compared with US suburban dynamics, despite the proclaimed exceptionality of the Parisian case study. (a) Anglo-american concepts and theories describing suburbs have been widely adopted to analyze suburbs in the Paris region, and rather unquestioned, such as boomburbs, edge-cities, gated communities, a terminology especially used to demonstrate the theories of fragmentation, polarization, privatization and secession of suburban areas [61–63]. Therefore, (b) a common oversimplification is to analyze the Paris region as a counter model to American cities, and a model of the supposed hallmarks of European cities: post-industrial transition, high unemployment, high level of skilled workers, higher densities, lower levels of social segregation, lower spatial mismatch, better public transportation [64]. In the same movement, Paris has also been compared to other metropolitan areas, focusing on two major standpoints: on the one hand similar trends towards gentrification and suburbanization; and on the other poor job accessibility, exposure to the negative externalities of concentrated poverty in deprived neighborhoods. (c) Such views have opposed outer suburbs as an homogeneous middle class, to gentrified city centers and over-segregated *banlieues* of public housing blocks, and have been criticized as an uncritical transfer and overreach of the Anglo-American concepts of ghettos [49], americanization of suburbs [65], fragmentation and secessionist attitudes [66, 67]. This is not only a media representation, as social scientists contributed to this shift in emphasis from socioeconomic characteristics, to an ethnoracial characteristics, thereby “validating the relevance of the US case as a point of comparison” [58]. Interestingly, recent work however pushes for more nuanced analysis of middle-class diversity and social mixing in segregation patterns [57, 60]

Meaning, Paris as a case-study is simultaneously and ambiguously used to both confirm and contradict some general hypothesis about global urbanization. This is even more true when discussing the typologies of suburbanization. In different comparative analyses, the Paris region is used to document variegated categories of suburbanism:

- a region characterized by an early sprawl of both bourgeois and blue-collar industrial suburbs [68], and influenced by the English model of single family-home suburbanization;
- a core city surrounded by satellites new towns produced in strong state-led planning and densification efforts [69];
- and also an historical example of an early americanization of residential landscape produced by developers [70, 71];
- an example of maximum fragmentation of local land policies, *nimbysm* and socioeconomic selection based on exclusiveness, supported by the small size of local government bodies, the communes [67], that fits well the evolutions of governance and privatization identified for instance by Keil and Young [72];
- an entanglement of different models and references that is also compared to and analyzed according to the *Zwischendadt* theories, literaly “in-between”, and the *città diffusa* models of “concentrated deconcentration” [72, 73];
- That last stage of suburbanization in France is also often analyzed as being post-suburban, a term that defines “in terms of inner suburban population loss and relative income decline, suburban employment increase, suburban out-commuting reduction, exurban population and income increase, and farmland conversion” [74]. “Post-suburban” describes the state of suburbanization in many countries [75, 76], although Paris metropolitan region is often *also* viewed as a counter model, insisting on the denser fabric of post-suburbanization in France and Paris region and a slower transformation of the monocentric structure of metropolitan areas [69].

In short, an overflow of empirical analysis describes the suburbs of Paris as a unique case study, and this paper may well be just another one. But this highlights the requirement to engage in a critical analysis of suburbs as both a products, economical and social processes.

Characterizing social change and the effect of the production regime on the property market and inequalities can be one way to do so. Taking transactions seriously is a means to study suburbs as an owner-occupied housing market, under the assumption that purchasing a property is a total social act [77]. This social act aligns the several dimensions of households’ income, assets and equity; their self perceptions and life aspirations; their lifestyles; their positions as a socio-occupational categories; their strategies and constraints in terms of preferential location and place of work; their abilities to fulfill the requirements of the financial and banking systems to get a loan approved, in a very normative way [7].

### Current debates in Paris ’burbs and research hypothesis

To do so, we argue it is critical to get a better understanding of the shape and dynamics of inequalities in the outer-suburbs of Paris, by the means of exploring the actual role that typical suburban single family homes, residential estates and subdivisions, have in property ownership, for instance on upward or downward mobilities [78], for two main reasons:

- First, in recent social and political science debates in France, some authors tend to reify single family home suburbs (and small towns) as segregated peripheries of the lower-middle class, described as isolated from metropolitan major financial centers, employment, and left aside by public policies, urban renewal and state-led provision of services [79]. Such analyses tend to provide the media and policymakers with a simplistic description of suburban residents as far-right voters, low-wage workers, and anxious, car-dependent property owners [80]. These views are based on spatial correlations between outer suburbs, sub-rural areas and far-right voters, although the ecological fallacy of this line of interpretation has been clearly demonstrated [81], because of complex and changing patterns of spatial segregation in suburbs. It is widely admitted that socioeconomic segregation in the Paris metropolitan region is still structured by a class-based segregation between executives, managers and higher-order management neighborhoods, and workers, i.e. the inertia of the 19e century divide [52, 82, 83]. This bipolar divide has been however rearranged with the rise of salaried employees (29,5% of the active population), intermediate occupations, the decline of blue collars (now 16,5%) and the restructuring of employment (part-time, unemployment, etc), as thoroughly studied by Préteceille [52]. Such a restructuring also fuels a debate between the contenders of social polarization versus professionalization [52, 84, 85] on the one hand, and increased inequalities because of income and asset-capitalization [86] on the other. We contend both interpretation are not mutually exclusive.
  *To better inform the debate, this paper elaborates on socio-occupational categories and property values, in an asset-based welfare system. We assume (hypothesis #1) that professionalization (a rise of executives, intermediate occupation and salaried employees, as defined in public statistics categories) well describes the dynamics of property ownership in suburbs, and that local trajectories in price appreciation are critical in understanding the different regimes of wealth accumulation for suburban households. Property prices, therefore, directly affect social and spatial inequalities of residents and buyers in a stratified market, and also intensify inequalities through asset-capitalization (hypothesis #2)*.
- Second, a strong public policy emphasis has been set on integrating city centers and suburban areas, under a reincorporation of metropolitan governmental bodies after 2016, but this reform explicitly excluded outer suburbs, because of their specific spatial and socioeconomic patterns, set aside from the rest of the core agglomeration [87–89]. This theoretical problem of the explicit linkages and dependance between outer-suburbs and the central agglomeration of Paris has however been preeminent in the literature.
  Suburbs have long been regarded as the locus for the lower middle class [90], excluded from more central locations by the high cost of housing. However, some recent studies have sought to show the greatest diversity of population dynamics, in terms of age, class, [22, 83, 91], or origin [92], as well as the variegated forms of political and social engagement [93]. To some extent, suburbanization in France is mostly seen as a movement of residential loosening that cannot be easily compared to the edgeless city [94]. Urban centers, however, have remained major places of employment; in 2010, suburban areas as a whole account for 12% of jobs whereas a quarter of employees are suburbanites. In the early 2010s, the average size of suburban municipalities was 820 inhabitants according to E. Charmes [67], and an estimated 30% of the population lives in statistical suburban areas. This growing discrepancy between place of residence and place of work feeds an intense growth of commuting trips between centers and peripheries. A certain level of job diversification has been seen beyond residential services [95], as well as a growing trend towards sub-centering in connection with the emergence of secondary job centers [96, 97]. Data show that the suburbs of Paris indeed face similar problems as other large metropolitan areas. Distances separating jobs from housing have been increasing, public transit systems are efficient within the very heart of the city but most trips connecting suburbs to suburbs are difficult to achieve within a reasonable amount of time, this having detrimental effect especially on low-skilled workers with poor job accessibility [64], although car-dependance effects on segregation are found to be mitigated by residential mobility [95]. Residential mobility is however impaired by the lack of affordable housing [98].
  To some extent, this paper remains in a tradition that consists in exclusively focusing on the outer ring of suburbs. By doing so, we aim at clarifying the effects of a tension between two interpretative trends, either of suburbs as a market being fueled by sprawl and social homogenization; or whether the maturation of suburbs yields a stronger diversification of socioeconomic profiles due to commuting patterns, sub-centering and locational strategies or constraints. *From this second standpoint, we assume that sub-centering and maturity of suburbanization should result in a structured and diversified pattern of homeownership segregation between the different social classes (hypothesis #3)*.

## Methodology: An empirical analysis of transactions in Paris suburban areas

The case study for this paper is located in the western part of the greater Paris region (Ile-de-France), an administrative region of 12,2 millions inhabitants (19% of the total population of metropolitan France). Housing has been characterized since the 1990s by continuous tensions on housing markets: according to public data from INSEE [98], 49.7% are occupied by renters, and 26,6% of the total housing stock are single family homes. The price index has been multiplied by a factor two for apartments between 1997 and 2017; and by 3,5 for homes. We analyzed in this research the submarket of single-family detached suburban homes, *i.e*. a typical component of suburban submarkets usually found in housing estates and tract-housing developments: data are provided for the outer rings of the greater Paris region, the 4 administrative districts (départements) of Yvelines, Val-d’Oise, Seine-et-Marne, and Essonne, a sub-region of 5,438,000 inhabitants in 2013, among which more than half of the population (51,6%) lived in individual housing units, and 60% were owner-occupied dwellings, according national statistics (INSEE) [98]. Individual housing has been produced under two different statistical categories, “Individuel” (Single detached homes), and master-planned tract housing: data describing development permits show that both types represent an outstanding majority of properties built in the outer crown of Paris between 1999 and 2011 [99].

### A geographical approach of the dynamics of prices and inequalities

To explicitly link the dynamics of property prices and the dynamics of inequalities on the market, we analyzed transactions using the characterization of sellers and buyers by socio-occupational categories in an owner-driven market. This strategy stems from the limits of the commonly stylized relationships between inequalities and housing prices. Many analyses of economic inequalities are based on the fact that inequalities and asset capitalization between households depend on housing value inflation within a crisis of affordability. However, from a methodological point of view, most of the standard approaches to housing markets assume that large parts of the differences between real estate values depend upon social and urban parameters and the socioeconomic composition of the neighborhood, *i.e*. inequalities and segregation.

Economic geographers mainly approach the issue of price through the hegemonic framework of econometrics, narrowing down the issue to control dependent and independent variables in modeling housing market segments. The immense body of work from spatial econometrics and housing segmentation derive from neo-classical models, which tend to explain property valuations through the mixed effects of fixed characteristics and spatial attributes [100]. Indeed, standard econometrics assign a value to a typical good, according to the hedonic attributes of the property (consent to pay for each of the attributes), under the hypothesis that sellers and buyers agree on a market price for the attributes. This is usually performed by the means of a regression model, explanatory variables derived from the attributes of the properties, characteristics of the surroundings (i.e. social and natural environment), and information regarding accessibility or locational characteristics [101]. Classical approaches focus on price formation more than on a detailed geography of property price and the actual mapping and representation of market dynamics. Information on the dynamic geography and socioeconomic profiles of sellers and buyers has therefore been overlooked in the literature.

Consequently, the explicit understanding of space is not always properly handled by the models: much of the research effort has been directed to the definition of submarket segments described by typical goods, and correcting or controlling for spatial autocorrelation problems in price determination, for which many modeling implementation have been tested [102, 103]. The need for better spatial analysis in hedonic price modeling in contextualizing the housing markets and its spatial interactions is often acknowledged [104]. But some scholars argue that space and distance are inadequate explanatory variable, some exploring the multilevel interactions of amenities with price according to distance in order to better account for geographically nested effects and scalar interactions [105, 106]; while others contend the need to radically over-simplify the problem of autocorrelation and consider spatial coordinates (x and y) as explanatory variables [107]. Sophisticated econometric approaches have also been tested to better include the variegated specifications of externalities resulting from neighborhood effects, street effects and locational effects. These are functions implemented with discrete variables constructed on distance thresholds from schools, parks [106], light-rail and trams [108] or urban renewal districts [109]. Such models also handle externalities e.g. the perception of environmental assets or noise [110]. This research have contributed to contextualize the effect of distance on property pricing. Elaborating a theory of price and spatial interaction however requires a better and explicit understanding of exogenous and relational socio-spatial interactions that interfere at many nested scalar effects.

Hedonic pricing focus on explaining price formation rather than on a detailed geography of property price and its mapping and representation. Though now implementing many refinements, this approach relies mostly upon the concept of equilibrium and pays little attention to prices dynamics. Price dynamics are indeed a geographical problem because of the remarkable catching up and convergence processes that occur between neighborhoods explained by spatially displaced demand, a dynamic observed for instance in London [85], and Paris [111], that strongly supports rent gap theory [112]. The rents extracted from urban locations convey the assumption that house price inflation could not be reduced to the invisible hand of supply and demand governing price dynamics. For instance a study examines the dynamics of price due to market leading market lagging phenomenon, by the means of a cross-correlation matrix representing price linkage between different areas in the UK housing-market spatially indexed time-series [113]. Hence, well-known phenomena related to the dynamic of property appreciation or decay have to be studied as spatial dynamics. Furthermore, it has been shown that housing price inflation does not necessarily involve a contraction of demand [114], nor does increased supply imply depreciation [115]. The reasons for this is that urban land is embedded in a system of value production and capture through which social, political and property relations of capitalism are intermediated [116], that also disconnects value from the market theoretical equilibrium. Some scholars therefore conceptualized real estate as other forms of capital and commodity [117], a means to advance the uncoupling of housing rent from land rent. Such uncoupling matters for the geography of real estate markets, as stock properties do not have to include increased costs of production (i.e. land rent), and prices of properties increase or devaluate while properties themselves do not upgrade or downgrade [118].

This problem highlights the needs for a more spatial approach [9], as well as the development of analytical tools to model, visualize, and explain the evolution and distribution of transactions, prices and accumulation across the differentiated spaces of the urban fabric. To do so, we test interpolation and visualization methods that rely on an explicit function of distance (travel-time), within suburban homogeneous (single-family homes in subdivisions), although spatially fragmented submarkets.

### Spatial transactions time series

Individual transactions for the whole metropolitan region of Paris were obtained from the Chamber of the Notaries—Paris Notaire Service (PNS), a commercial provider (for details on data provision, see S1 Methodological Appendix). This database contains a sample of transactions for the region and its suburbs, within the administrative limits of Ile-de-France (1 million rows), covering a 16 year timeframe: 1996, 1999, 2003, then every year from 2004 to 2012. All transactions are geographically indexed, with the address, the parcel number, latitude and longitude. From this dataset, we have extracted the single-family detached suburban homes (*pavillon*), a typical component of suburban submarkets usually found in housing estates and tract-housing developments (152,449 records), in 815 suburban municipalities of the four outer ring districts (Yvelines, Essonne, Val-de-Marne, Val-d’Oise).

**Fig 1:**
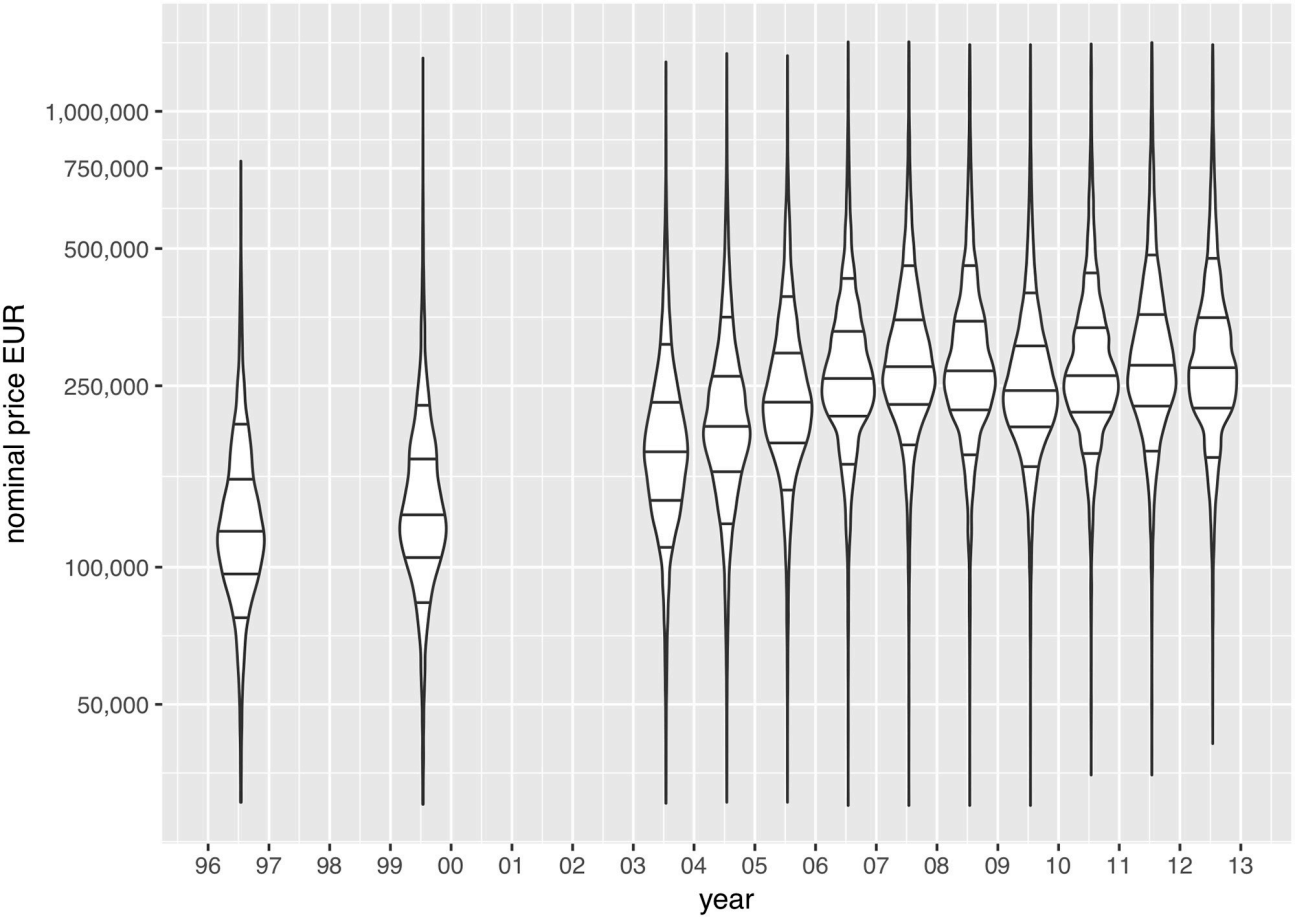
Univariate distribution of house values, in Euros, 1996-2012. Violin plots represent kernel density estimates. Thresholds defined as 1st decile, first quartile, median, third quartiles and 9th decile; price scale, *log*_10_. For breakdown of price trends by socio-occupational categories, *cf*. S1 Fig.

**Fig 2:**
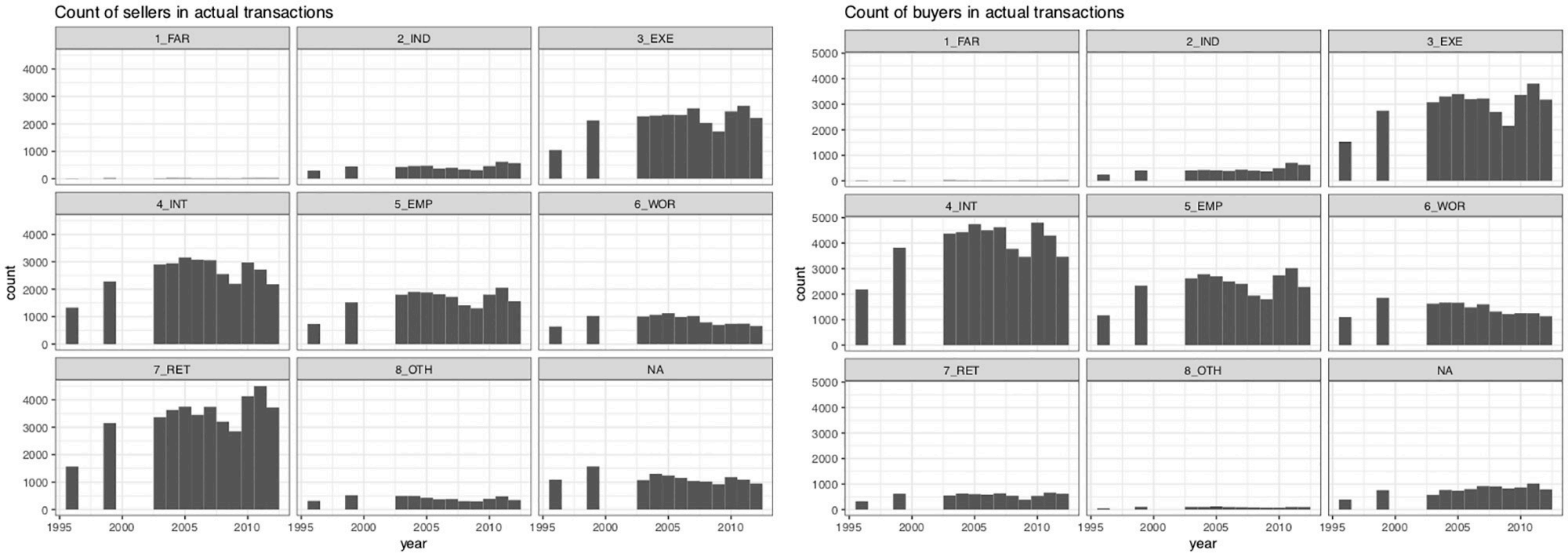
The average profile of sellers and buyers, 1996-2012 (Frequencies). 1 FAR: Farmer; 2 IND Craftsmen, business owners, independent workers; 3 EXE: professionals, executives, academics, engineers; 4 INT Intermediary occupations;5 EMP Salaried Employees; 6 WOR Workers; 7 RET retirees; 8 OTH Other and unoccupied. Other categories e.g. Real Estate Professional and REITS set aside in the analysis (NA). Source: BIEN Database, PNS, 2012.

All records contain information on property amenities and pricing (Fig 1), but also a series of understudied variables on sellers and buyers, such as age, sex, socio-economic status, national origin, place of residence, and mortgage. The main variables used in this study are property price and occupation of sellers and buyers when the property is sold or purchased by an individual (Fig 2), set aside are real-estate investment trusts (REITS) and real-estate professionals. Residential markets in France, especially in suburbs, have not been structured yet by institutional investors and investment funds [119]. Regarding the price, the aim being to distinguish between the various local patterns of appreciation and depreciation, we adopt the nominal price of properties as an indicator of housing price inflation. In a national context where price inflation is decoupled from macro-economic fundamentals (slow economic growth and low inflation in France during the last decades) [1], we are more interested in the unequal geography of nominal price dynamics, from which stems affordability issues for households and increased price to income ratios.

**Fig 3:**
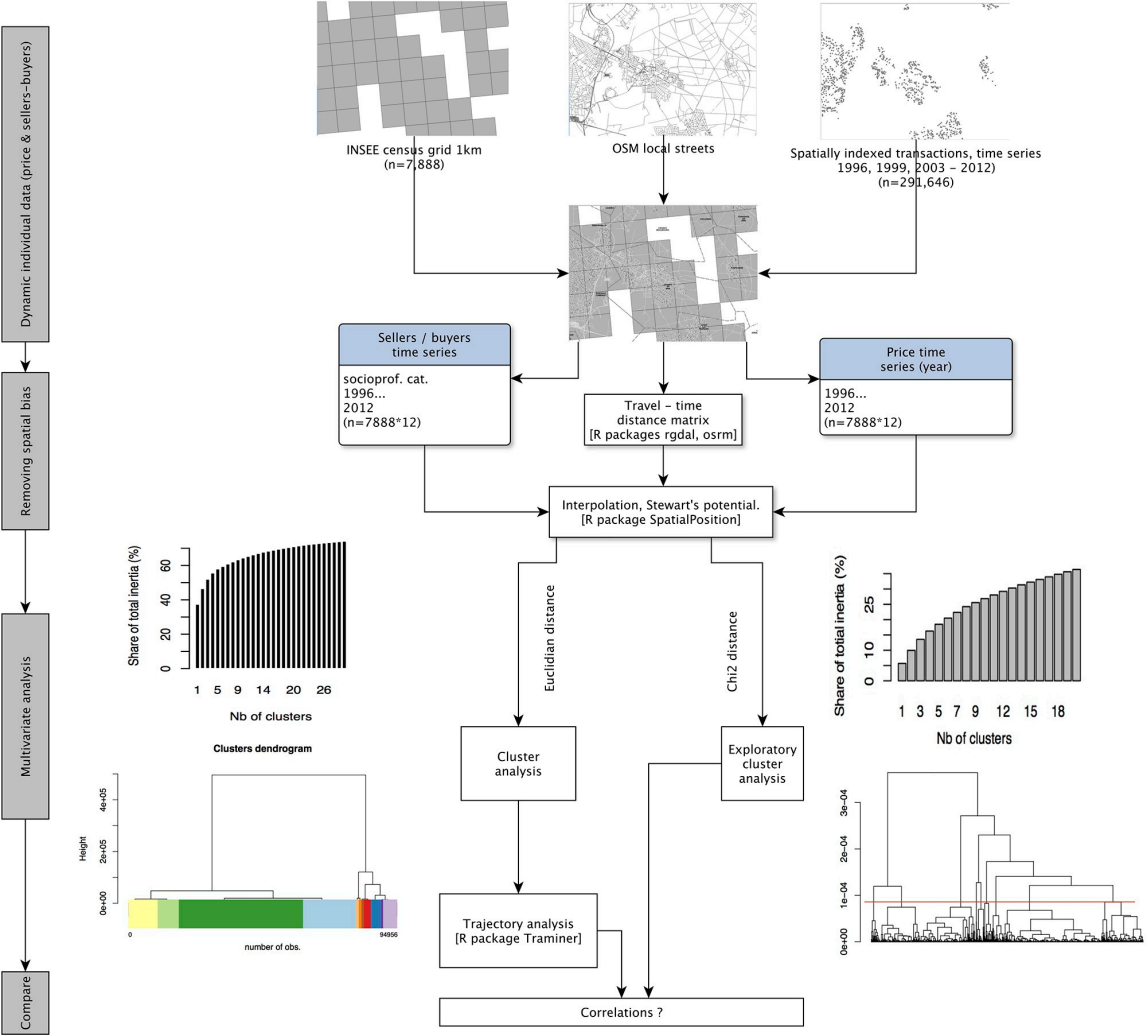
Data-flow diagram.

Although they are public records, transaction data are considered in France a proprietary database, distributed to researchers by PNS as a commercial product and subject to restrictions for the dissemination of results. Given the cost of the database for public research institutions, only a limited number of years and a sample of transaction have been acquired, considering the scholarly work that has already been published using datasets covering the 1993-2006 period for the Paris region [11, 111], and some exploratory work recently conducted on suburban market data [12]. Data flow is described in (Fig 3). Detailed methodology regarding data selection and sampling is provided in S1 Methodological Appendix.

### … matched to a census grid using a travel-time matrix

For spatial analysis purpose, two final issues had to be dealt with: the weakness of samples when matched with small local geographies, and the fragmented structure of the built environment, made of subdivisions, large tract housing development, but also detached houses scattered in semi-rural landscapes. To offset these limitations, a combination of a suitable grid and techniques of interpolation of point data was used.

**Fig 4:**
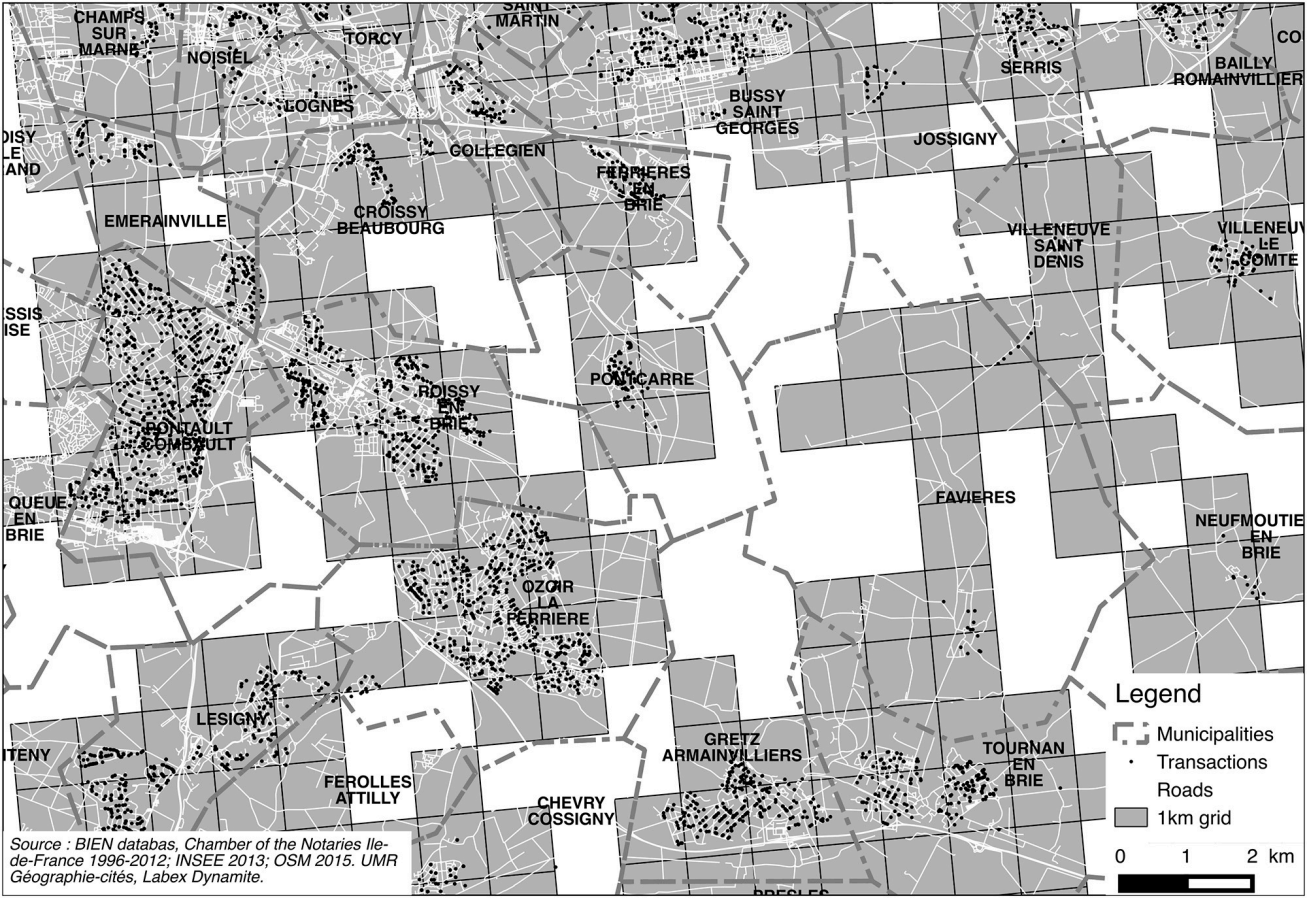
Sample of transactions, populated areas, road segments and 1 km grid compared. East-south-east of Paris (Val-de-Marne), nearby Marne-la-Vallée. Disneyland Paris is located north of this map. Source: OpenStreetMap 2016; INSEE 2016; BIEN Database, PNS, 2012.

Because of requirements regarding the confidentiality of individual transactions, it was impossible to use aggregates of less than 5 transactions. Given the spatial distribution of transactions, it is challenging to find any suitable geography that will allow us to render finer grain local dynamics and maintain the requirement for aggregating transactions. Given the spatial fragmentation of transactions in the outer peripheries, the problem remains when using larger spatial units, such as municipalities. The main analysis was conducted at a 1km-cell grid level as provided by the French census institute (INSEE) for local analysis. Fig 4 also highlights why the geography of municipal boundaries usually used to map property prices are inadequate in many cases in suburbia as it does not fit the actual geography of suburbanization and neighborhoods made of a mix of close-knit subdivisions and scattered countryside homes. As discussed in the literature, amenities, exclusivity, club realm and locational rent strongly interact in producing socioeconomic homogeneity at the neighborhood or subdivision level [120–123]. This grid combines three main advantages for a study of suburban areas (details provided in S1 Methodological Appendix):

1. This is an appropriate spatial proxy for homogeneous areas matching the fragmented suburban built environment, as secondary street segments generally define local submarkets [124–126].
2. The grid is an appropriate proxy for homogeneous areas matching the fragmented suburban built environment. It fits the spatial patterns of urbanization, and does not impute a value to areas that have no values, or no potential buyers or sellers. It is also consistent with regulations on data and statistical secrecy.
3. Matching the “real estate agent paradigm” (*i.e*. assessing price of a property with reference to nearby similar properties), the interpolation uses a travel-time matrix between cell centroids. The computed values will be therefore imputed to the closest cells, making two nearby properties more likely to be priced equally (spatial interaction hypothesis) if cells are connected by local streets.

### An interpolation of discrete socioeconomic data

The methodology also relies on an interpolation of discrete socioeconomic phenomena: prices and sellers / buyers occupational categories. Interpolation is a classical problem: in many cases, the problem consists in mapping or visualizing a continuous surface (temperature, wind) where the phenomenon can be accurately estimated in all points, with a small number of actual measures. But the usual methods of spatial interpolation (e.g. triangulation, krigging, all based on sampling theories and inferential statistics) are unfit in the specific case of discrete phenomena [127]. Some solutions have been implemented on real-estate advertising websites, that deal with the problem of generalizing the information from transactions in a neighborhood. For instance, if we consider the industry’s solution to “real world problems”, the mapping engine of a website such as Meilleursagents.com renders a continuous surface of prices at a smaller scale, but when zooming on a specific suburban neighborhood either averages the property values at the municipal geographical level, or individuate the information by mixing different geographies on the same map: parcel / neighborhoods and municipal levels, depending on the significance of samples of sold properties (for example, in Versailles and its vicinity: http://www.meilleursagents.com/prix-immobilier/versailles-78000/).

We propose an alternative approach that computes a synthetic value based on distance and weight of the observed population, as initially proposed by Stewart [128] for an analysis of the distribution of student population and catchment areas of American Universities, and more recently applied for socioeconomic phenomena [129]. We infer that property markets are discrete social data, similar to Tobler’s hypothesis [130]: a potential price for a specific location is a function of distance to nearby similar transactions, and also a function of the number of properties available, turnover and realized transactions. This method removes spatial bias, resolving the Modifiable Areal Unit Problem (MAUP), as demonstrated for demographic indices in Europe [127, 131]. We elaborate on Grasland’s framework for spatial analysis of social facts [129], based on Tobler’s first law of geography [132] and Stouffer’s intervening opportunities [133], justifying to use Stewart’s potential [128] for the spatial interpolation of social discrete data (details in S1 Methodological Appendix).

We apply Stewart’s potential to house price, and also to the number of sellers and buyers of each occupational category (Fig 2), using the *SpatialPosition R* package [134, 135]. The potential of population is generally defined as a stock of population weighted by distance:

$$\begin{array}{c} A_{i}=\sum_{j=1}^{n}O_{j}f\left(d_{ij}\right) \end{array}$$ (1)

where *A*_*i*_ the potential of *i*, *O*_*i*_ the stock of population at *j*, *f*(*d*_*ij*_) a negative function of distance, generally a power or exponential curve. Function parameters have been estimated by the means of semi-variograms, *i.e*. an estimation of the spatial variability (the variance of a parameter considering the lag or distance between pairs of datapoints). We have elected to implement a Pareto function, with a span of 10 minutes (travel-time by street network) and a *β* parameter of 0.27; *α* is defined as the distance where the density of probability of the spatial interaction function equals 0.5, as documented in the *SpatialPosition R* package [135].

$$\begin{array}{c} f\left(d_{ij}\right)={\left(1+\alpha .d_{ij}\right)}^{-\beta } \end{array}$$ (2)

with

$$\begin{array}{c} \alpha =\log\left(2\right)/span^{\beta } \end{array}$$ (3)

**Fig 5:**
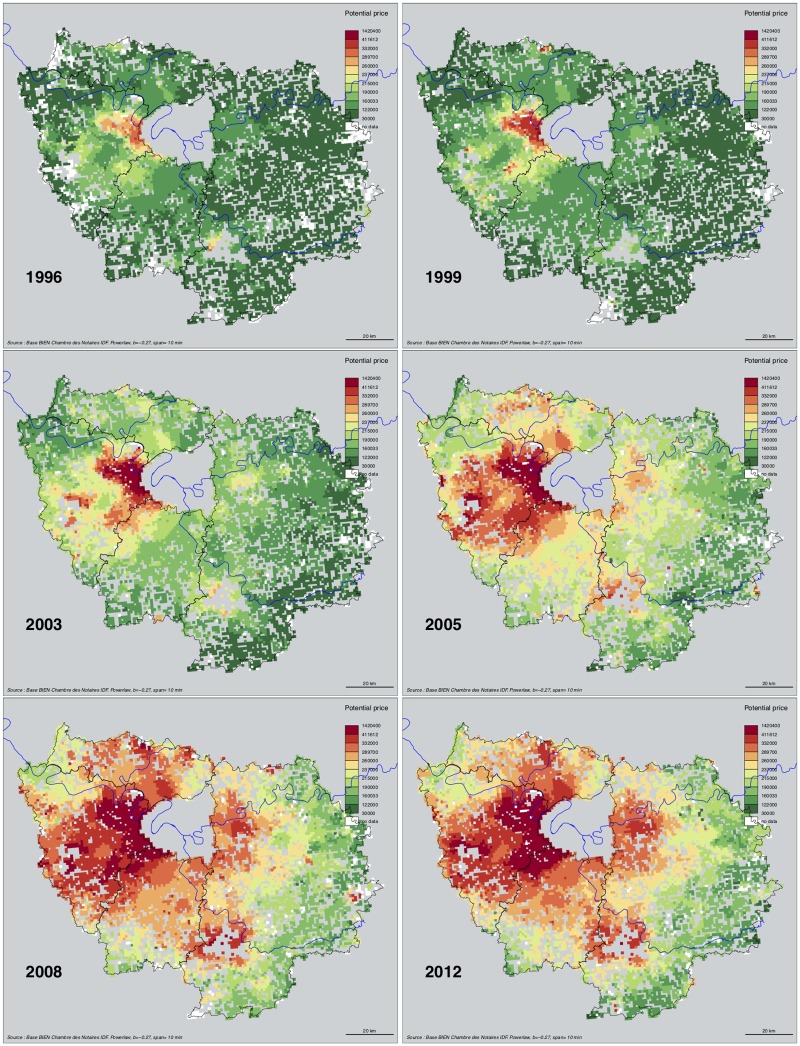
Potential price (EUR) of single family homes, within 10 min. neighborhoods. Selected years. Source: BIEN Database, Chambre des Notaires IDF, 2012. Interpolation with R *SpatialPosition R* package, Powerlaw *β* = −0.27, span = 10. Author: R. Le Goix, 2016, UMR Géographie-cités, Labex Dynamite.

The computation of the price potential follows a two step procedure: first, the potential for price is computed as the potential total value in a cell (*Pp*); then, the potential for the number of transactions (*Pt*) in a given cell is computed. The potential house price for a given cell equals to *Pp*/*Pt*, and then matched to a grid and mapped (Fig 5). To characterize change and local patterns of inflation, we apply a cluster analysis based on property prices. Applied to the number of sellers or buyers of each category, Stewart’s potential is computed as for a stock of population *O*.

### Using annual sellers-buyers balance to analyze neighborhood change

With gridded interpolated variables, we perform a multivariate analysis to cluster neighborhood change, as outlined in Fig 3. The same interpolation technique is also applied to determine the potential number of transactions within a 10 min. neighborhood for each category of sellers and buyers, for each given year. We apply the following steps to analyze changing balances between sellers and buyers for each category, and to compare the transitions and sequences of neighborhoods, revealing the differences in the dynamics of neighborhoods.

**Fig 6:**
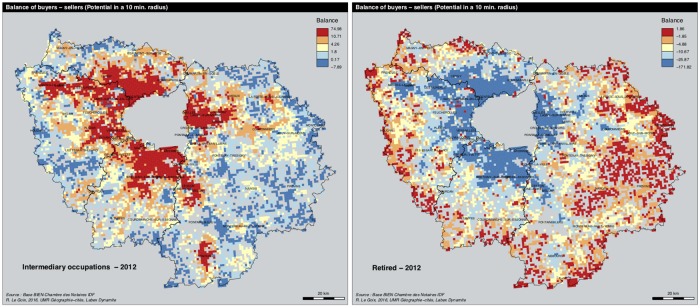
Sellers—Buyers balance in 2012. A: Intermediary occupations. B: retirees. Source: BIEN Database, Chambre des Notaires IDF, 2012. Author: R. Le Goix, 2016, UMR Géographie-cités, Labex Dynamite.

1. We use the information describing the sellers and buyers’ socio-occupational category: workers, intermediate occupation, salaried employees, executives, independent workers and retirees. The data do not allow us to analyze upward or downward mobility of households *per se*: the dataset describes the socio-occupational category of households (Fig 2). To analyze social change, we focus on the local balance between sellers and buyers in each grid cell, for each category, for each year. By doing so, we adopt a design that assumes that segregation stems from the balance between groups moving from one place to another. As an example, Fig 6 shows the dynamics for two main categories of actors. Intermediary occupations are more active as buyers than sellers in the inner part of the region, whereas retirees show an exact opposite trend: in 2012, they are more likely to move and buy properties in the outskirts and exurbs, leaving the mature suburbs of the first rings. We also analyzed the matrix of correlations between the proportion of buyers and sellers of each category, showing the main effect of inertia (*cf*. correlation matrix in S2 Fig). Because of the structure of exclusive trends (executives selling to executives; workers and employees more likely to sell to workers and employees), we deem that the balance between transactions are good indicators of local change.
2. Second, using sellers-buyers balance by categories as input variables describing each cell, a cluster analysis (ward method, euclidian distance) describes the categories for each neighborhood at each given date, assuming the different socio-occupational net balances are significant in analyzing local trends.
3. We finally elaborated on a sequence analysis method to sort out neighborhood change in US metropolitan areas [136, 137]. We analyzed the longitudinal categorical sequences of neighborhoods, applying sequential pattern mining procedures. For this we used the R TraMiner package algorithms [138] to sort out and describe the successive sequences of states for each neighborhoods, for each given year. The method sorts out the most frequent permutations found in the sequence of states for each neighborhood, so as to analyze local change as sequences and permutation between different states: for instance when a new suburban neighborhoods evolves and become an aging declining suburb. A clustering method was applied to aggregate the sequences into a number of groups. For that purpose we used the *agnes* function (Cluster library) of the TraMiner package.

## Results and discussion

The results of the study provide a thorough spatial analysis of property ownership change, property value trajectories and permutations of dwellers’ occupational categories as components of neighborhood change between 1996 and 2012. First, we present and discuss the results for the dynamics of property values over time, in order to better contextualize the unequal home value inflation in which transactions are taking place. Second, the main trajectories of property value growth and decline are summarized by the means of an exploratory cluster analysis. Third, we classify the cells describing neighborhoods by sellers-buyers balance for each category at each date, and analyze the longitudinal categorical sequences of neighborhoods between 1996 and 2012. We finally analyze the correlation between price dynamics and longitudinal sequences of neighborhoods. When appropriate, results are discussed with the literature within this section.

### Suburban home value dynamics: Generalized but unequal inflation

Regarding home price inflation between 1996 and 2012, data show an interesting combination of two trends: a constant appreciation in the entire region, and an apparent homogenization of prices towards the higher brackets of property values above 200,000 Euros (Fig 5. For an animated visualization *cf*. S3 Fig).

**Fig 7:**
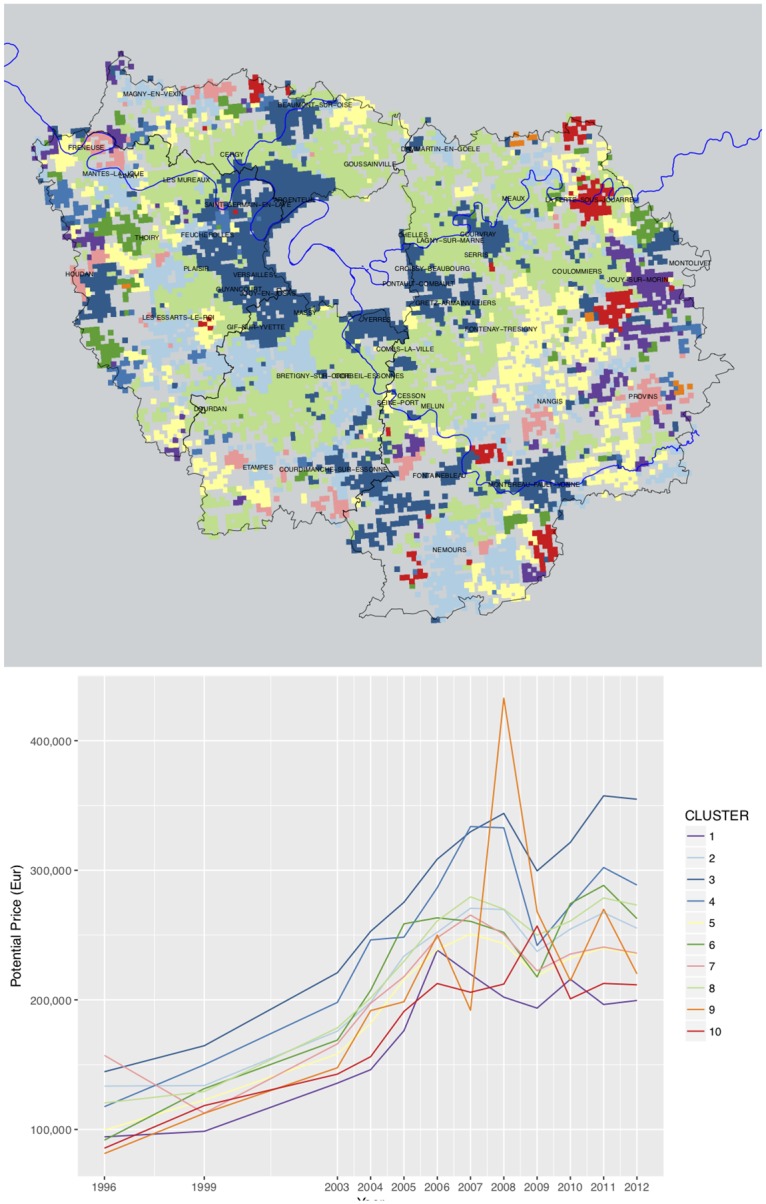
Typology of house price dynamics, 1996-2012. A: Map of clusters. B: Profiles of average home values by clusters. HCA of potential prices, *χ*^2^ metric, Ward method, *r*^2^ = 25%. Source: BIEN Database, Chambre des Notaires IDF, 2012. Author: R. Le Goix, 2017, UMR Géographie-cités, Labex Dynamite.

The spatial patterns of price inflation showed mixed and heterogeneous tendencies. To explore the resulting dataset, we use gridded potential price for each year as variables into a *χ*^2^ metric hierarchical cluster analysis, that yields more detailed local tendencies than the univariate trends summarized by deciles and quartiles in Fig 1). Cluster profiles of price inflation are reported in Fig 7: blueish colors describe the trends of highest price brackets (above 300,000 euros), green colors corresponds to median price brackets, and reddish colors describes lowest price brackets. The general trend is that price inflation has affected almost every suburban context, but it has also increased spatial heterogeneity between neighborhoods. Inflation has also strengthened the unequal spatial structure of prices and the hierarchy of neighborhoods. The spatial structure of unequal price growth over time follows 7 distinct trajectories:

- First, the hierarchy of neighborhood price brackets was maintained and strengthened between 1996 and 2012. Data show an increased gap between lower brackets and upper brackets: the average price between cheapest neighborhoods and expensive suburban neighborhoods has increased, with an interval of 150,000 euros in 2012, against 50,000 euros in 1996, as shown between *clusters 1 & 3*, describing the extreme values of the price spectrum in purple and blue colors.
- In *cluster 3* more expensive high-end neighborhoods near Versailles, Saint-Germain-en-Laye, Fontainebleau, Coupvray better recover from the 2007-2008 crisis, compared to furthest neighborhoods similarly priced in 2007, but experiencing a substantial value gap after 2007 (*cluster 4*). Such neighborhoods are those that better control their local environment, land availability, and develop local strategies of territorial control, exclusion, and club economy, in order to protect local tidiness and social homogeneity, as discussed by Charmes in his essay on the clubbization of French Periurban municipalities [67]. Clubbization is classically viewed in terms of club spaces—spaces governed by “small publics”—that is, homeowners associations, and shareholders. In the French context, it derives from the governance structure of small municipalities, local bodies of government whose principles perfectly match those of the club economy, as a local organization managing the interest of its members, most notability by means of slow-growth policies and control of land use [67].
- We also notice a slower price appreciation in average price contexts, in *clusters 2, 5, 6, 7 and 8*. All have similar prices between 1999 and 2006, then each of these clusters follow disctinct trajectories after 2007. *Cluster 8* for instance describes areas of mature subdivisions in the outer-suburbs built between 1970 and 1990, such as Cergy, Les Mureaux on the West-side; Goussainville, Meaux on the North-East side; Lagny, Coulommiers on the East part of the region; Cesson, Melun to the South: although properties lost an average of 25,000 Euros during the crisis, the recovery was complete in 2011, up to 275,000 euros for a typical suburban tract home. This trend is almost similar to trajectories followed by neighborhoods in furthest location like in Thoiry (*cluster 6*), but the depreciation of values started earlier. In remote areas with a mix of exurban and rural settlement, as well as in small peripheral towns such as Provins and Houdan (*clusters 5 and 7*), very slowly but not entirely recover after 2007. Neighborhoods described by *cluster 2* (Nangis, Nemours, Magny, Etampes) however show an incomplete recovery of residential markets.
- Lower price brackets also tend to depreciate after 2007: data show a slow depreciation in lower priced neighborhoods (as in *clusters 1, 7, 9, 10*, see the purple, red, orange lines after 2006). *Cluster 1* shows a common pattern of absolute continuous depreciation starting with the global real-estate and financial crisis as soon as 2007, mostly in the far-eastern part of the region (Jouy-sur-Morin). In other cases, such as *cluster 10*, the depreciation started only after 2009, which describes remote exburban areas e.g. La-Ferté-sous-Jouare. This depreciation of lower-end neighborhoods even yields very volatile trends in the furthest peripheries in the lowest brackets of property prices after 2007 as in *cluster 9*, although outliers, rare and exceptional transactions may produce such volatile local trends. Such decreasing trends, although sometimes interpreted as a long awaited stabilization of markets and good news for affordability, are however inherently burdens that put indebted households at risk: their financial vulnerability will link the depreciation of assets and negative equity. Many local contexts follow such unsustainable patterns. These declining tendencies highlight the lack of sustainability of some remote suburban neighborhoods and subdivisions. Such trends inform how households in maturing and lower-end subdivisions may be trapped in place [139] by decreasing property values, which compromise their capacity for reinvestment because of devaluation and even negative equity; whereas other comment the depreciation and decline of furthest away subdivisions, rendered obsolete by increased energy costs that impacts the burden of daily commuting and shopping trips by car [140].

These first series of results show common grounds with trends identified in more central places: where generalized inflation has strengthened the unequal spatial structure of price and the hierarchy of neighborhoods. Suburban housing however shows a greater tendency to follow heterogeneous trends, compared to more central locations. The center of Paris and its inner suburbs are more likely to show more homogeneous prices because of inflation [11, 111], as well as in Marseille [9].

### A typology of sellers-buyers balance

We now examine the results of the typology of sellers-buyers balance to analyze neighborhood change in connection with the variegated local trajectories of inflation. The main goal of this typology derives from the overarching hypothesis of the research: rather than dualization (executives vs. workers), professionalization (a rise of executives, intermediate occupation and salaried employees) more accurately describes the dynamics of property ownership in suburbs, in close connection with local trajectories in price appreciation (H1).

**Fig 8:**
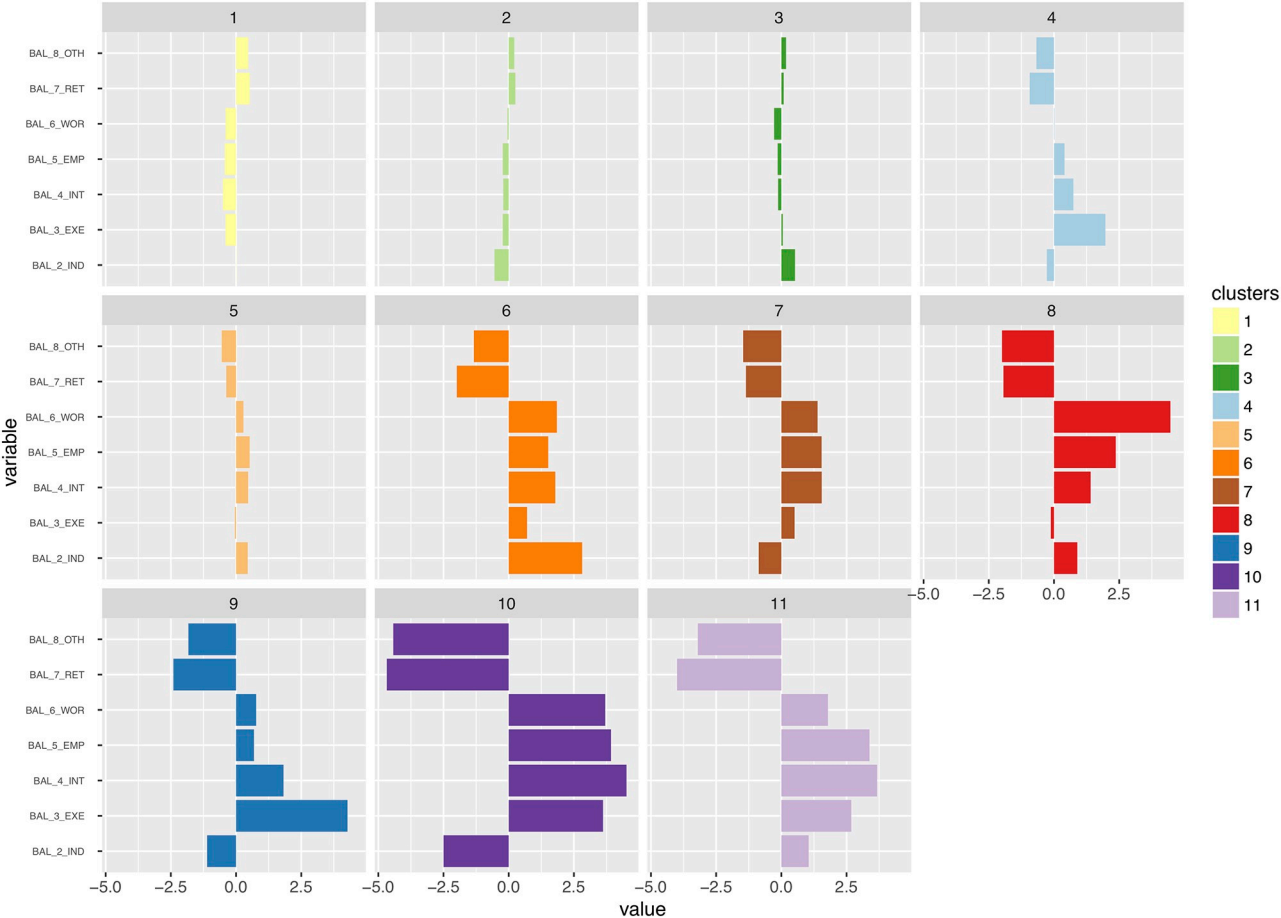
Typology of sellers and buyers balance, in single family homes in Ile-de-France, 1996-2012: Normalized z-scores of variables by clusters. Selected years. Source: BIEN Database, Chambre des Notaires IDF, 2012. HCA, euclidian distance, Ward method, N = 7888 cells * 12 years, solution with 11 clusters, *r*^2^ = 61%. 1 FAR: Farmer; 2 IND Craftsmen, business owners, independent workers; 3 EXE: professionals, executives, academics, engineers; 4 INT Intermediary occupations; 5 EMP Salaried Employees; 6 WOR Workers; 7 RET retirees; 8 OTH Other and unoccupied. Other categories e.g. Real Estate professionals and REITS set aside in the analysis (NA). Author: R. Le Goix, 2017, UMR Géographie-cités, Labex Dynamite.

**Fig 9:**
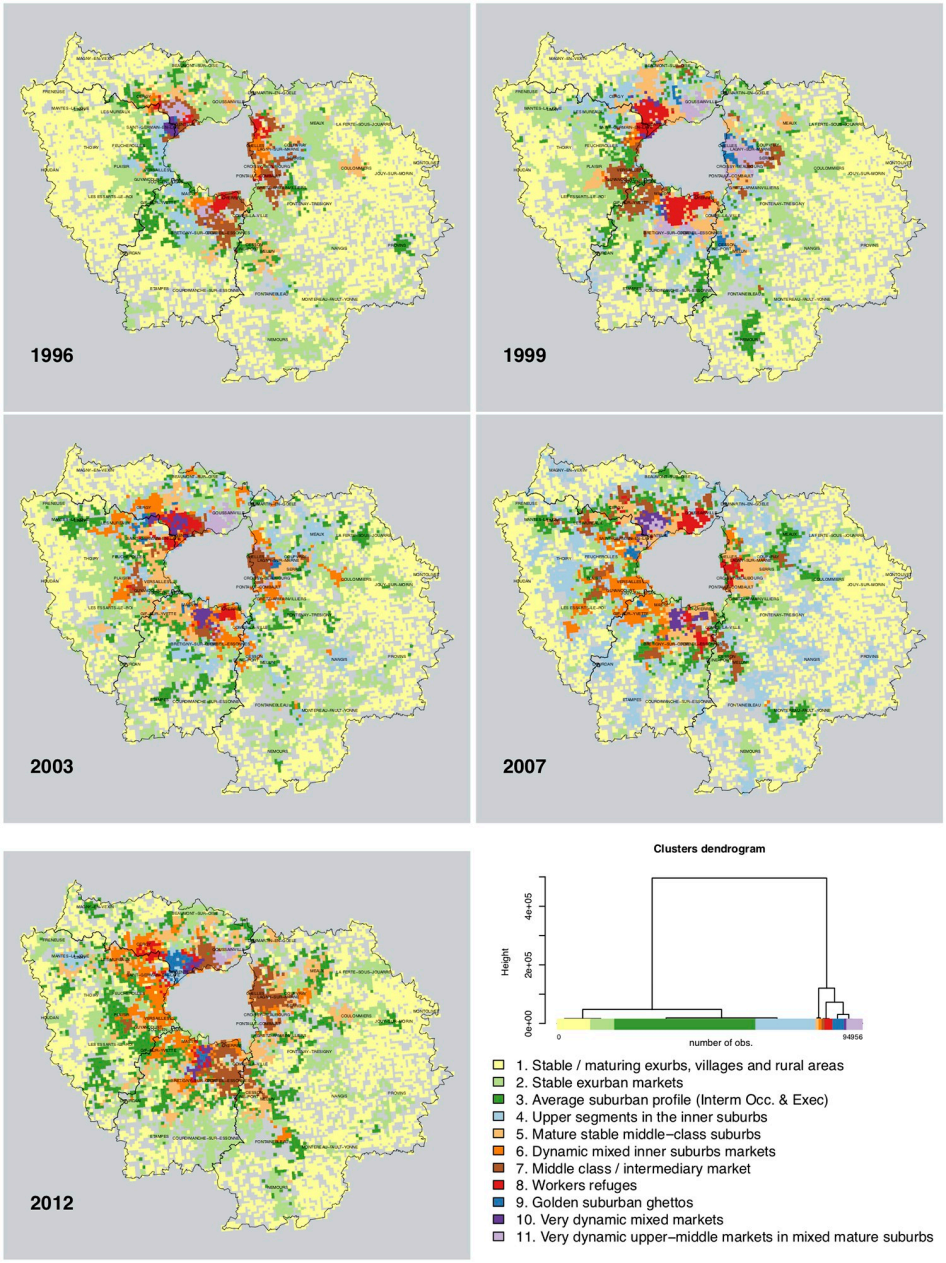
Typology of sellers and buyers balance, in single family homes in Ile-de-France, 1996-2012. Selected years. Source: BIEN Database, Chambre des Notaires IDF, 2012. HCA, euclidian distance, Ward method, N = 7888 cells * 12 years, solution with 11 clusters, *r*^2^ = 61%. Author: R. Le Goix, 2017, UMR Géographie-cités, Labex Dynamite.

The typology describing the states of each neighborhood for any given year derives from a multivariate analysis (hierarchical cluster analysis, euclidian distance, Ward method, N = 7888 cells * 12 years, solution with 11 clusters, *r*^2^ = 61%). Each cluster is described according to the z-scores (Fig 8) and mapped in grid cells (Fig 9). For an animated visualization of the typology, *cf*. S4 Fig.

The eleven clusters describe different stages of stability, maturity, or rapid change in neighborhoods. Each cluster, for any given year, describes the significant socio-occupational categories balance between sellers and buyers; clusters are interpreted as a momentum between sellers and buyers, *i.e*. a change in balance compared to the average profile described on Fig 2. A first series of clusters is overrepresented, as on the dendrogram on Fig 9, and are variations close to the average profiles. *Cluster 1* describes an average profile of neighborhoods, characterized by a complete stability of socioeconomic profiles across all socio-occupational categories: z-scores indicate a light trend of maturing population with more retirees moving in neighborhoods that can be described as stable and maturing exurbs, villages and rural areas with residential settlements made of scattered single family homes. This is the only spatial context in which retirees showed a positive balance: as in Fig 2, retirees were moving out of the Paris region, selling more than they bought. In exurban subdivisions and suburban areas, *Clusters 2 and 3* are variations in trends and magnitude of this average stable profile, characterized by the average dynamics described on Fig 2, *i.e*. intermediate occupations and executives having been the main actors with a positive balance as buyers. As on the maps, *cluster 2* best describes *stable exurban markets*, whereas *cluster 3* clearly delineates an average suburban profile where intermediate occupations and executives were leading actors on both selling and purchasing markets. Neutral z-scores demonstrate that these trends correspond to the regional average, with however less blue collar workers and relatively more craftsmen and business owners (2_IND) in *cluster 3*. In *Cluster 4*, executives moving in the areas are however overrepresented, all other variables remaining around the average profile.

In other neighborhoods, data show selective patterns of instability, more change specifically described by overrepresented socio-occupational categories, with moderate dynamics driven by intermediate occupations and to some extent independent workers and crafstmen, in mixed inner suburban markets, that were less attractive for executives, a group more likely to move out of these areas (*Clusters 5 & 6*). *Cluster 7* describes inner suburban neighborhoods with a steady influx of the middle-class (intermediary occupation and employees), exclusive of other categories.

Finally, in some areas, very rapid change has been produced by an overwhelming influx of some socio-occupational categories, mutually exclusive of others. *Cluster 8* is clearly characterized as the last refuge of blue collar workers in heavily deindustrialized region: these were the last neighborhoods in which the dynamics of workers as buyers superseded the dynamics of workers selling properties. *Cluster 9* is clearly defining places where social change was produced by a strong and significant balance favoring the arrival of professionals, executives, academic and engineers. It better defines places in which urban renewal, gentrification, and also the patterns of employment and transportation has made the housing stock more attractive to this social group. Often spatially associated with *cluster 4*, *cluster 9* is an avatar of the suburban golden ghetto. *Cluster 10* describes a very dynamic very mixed market: a common trend characterized by an overrepresentation of all categories: workers, salaried employees, intermediary occupations, professionals and executives, all with strong positive balances, against retirees, massively selling in this diverse active market. Such trends are likely to be found in areas of rapid change in the built environment (renewal and infill development). *Cluster 11* is a variation of *cluster 10*, with an overrepresentation of salaried employees and intermediate occupations as buyers more than as sellers.

This partition demonstrates the validity of hypothesis #1 (professionalization as a driver of change in socioeconomic segregation), as increased numbers of executives, intermediate occupation and salaried employees are predominant actors on the markets, as sellers, and also as buyers, the differentiated impact of these three categories being well circumscribed by the cluster analysis. Hypothesis#3 is also well supported by the analysis: sub-centering, deindustrialization and the maturity of suburbanization yield a very structured and highly diversified pattern of segregation. It is also clear from the comparison between prices (Fig 7) and the balance between sellers and buyers (Fig 9) that not only changes in socio-professional structure explain segregation, but households are sorted out between neighborhoods according to the variation of prices, the variegated patterns of asset capitalization in real estate value (Hypothesis#2). From this analysis, we conclude, with Preteceille [52, 58], Clerval and Delage [56], that the classical division white collars (executives / employees) *vs*. blue collars (workers) does not allow us to fully comprehend the social dynamics in the peripheries of Paris. This critique is also to be extended to the methodology employed in research on socioeconomic change, using classical segregation indices. Such indices were initially developed to study segregation patterns between racialized groups [141]. This method can be to some extent misleading when referring to dual patterns of socio-economic segregation, applied to a commonly used divide in the analysis of socioeconomic segregation patterns [13]: such research is classically constructed to juxtapose opposite groups on the socio-professional spectrum e.g. workers *vs*. executives [51, 79].

### Longitudinal categorical sequences of neighborhoods show an increased socioeconomic diversity

Given the aforementioned sellers-buyers typology, described for each given year, the last series of results derive from the sequencing of consecutive states for each neighborhood. We analyze how the socio-economic patterns of a neighborhood change over time with the succession of sellers moving out and buyers moving in.

**Fig 10:**
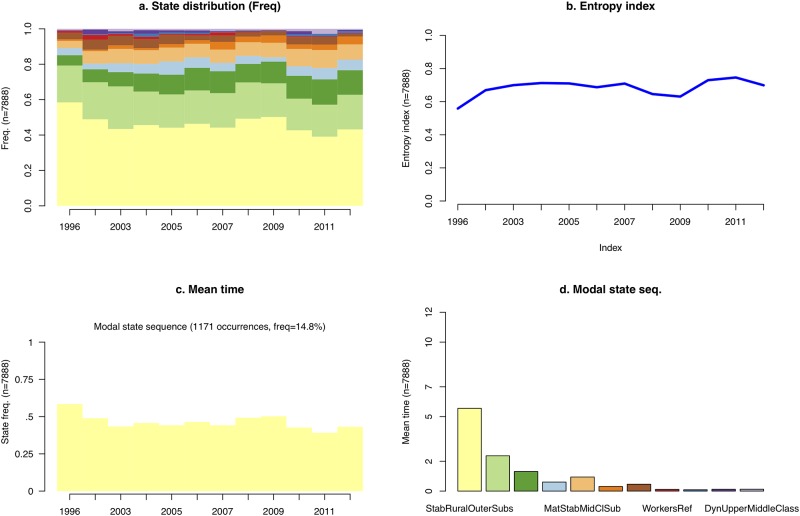
Descriptive statistics of the sellers-buyers typology sequence analysis of neighborhood change. Prepared with R package TraMiner 2.0-8. Source: BIEN Database, Chambre des Notaires IDF, 2012.

To do so, the R *TraMiner* package algorithms [138] were designed to sort out and describe in sequences the successive states of neighborhoods: this allows us to analyze local change as sequences and permutations between different states. To better map the trajectories of the sellers-buyers balance, we use this as an exploratory tool, adding values to the more static cartography (Fig 9 and animated version in Fig S4 Fig). The main results are described on Fig 10 with descriptive statistics on modal categories: data show that mostly rural and exurban categories *clusters 1 and 2*) are overrepresented, with an increased share over time of *cluster 3* (average suburban profile), *cluster 4* (upper segments in the inner suburbs) and *cluster 5* (mature stable middle class suburbs). An assessment of the relevance of such an analysis is provided by the entropy index on 10, which is a “measure of the diversity of states observed at the considered time point” [138]. We note that between 2007 and 2009, there was a decrease in diversity in the typology, followed after 2009 when the market recovered by a significant increase in diversity of state permutations. This can be interpreted as a result of market adaptations to the global 2007-08 real-estate and financial crisis. The crisis had lesser ramifications in France compared to other OECD countries, however, the results on the distribution of sellers and buyers has been a stabilization of the market with a lower level of variety in transactions in suburbs. The dynamics of the market, in terms of diversity of socioeconomic profiles and potential for local change, increased when the market recovered after 2009. This increased diversified pattern of segregation supports hypothesis #3.

**Fig 11:**
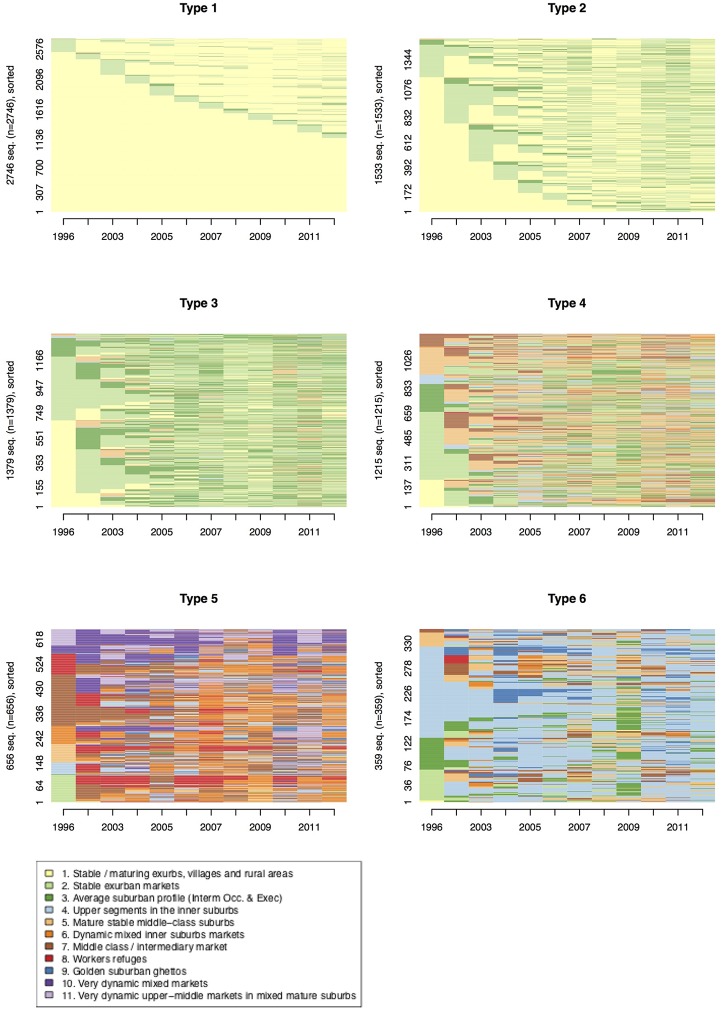
A typology of sequence analysis to explore sellers-buyers dynamics neighborhood change. Prepared with R package TraMiner 2.0-8. Source: BIEN Database, Chambre des Notaires IDF, 2012.

Finally, a last exploratory hierarchical clustering was also applied to the neighborhood sequencing. For exploratory analysis, the goal was to summarize the results of the sequence analysis and to aggregate the sequences into a reduced number of groups. Results, summarized into 6 clusters, as on Fig 11, account for 65% of the variance.

- A vast majority of exurbs and outer suburbs show extremely stable patterns over time between 1996 and 2012 (54% of sample). Stability in profiles is explained by a constant influx of retired persons moving in neighborhoods, in areas that can be described as stable and maturing exurbs, villages and rural areas with scattered estates and small subdivisions (*sequence type 1*, n = 2746). Exurbs *sequence type 2* (n = 1533) are also characterized by a more moderate influx of retirees, and by an average net inflow of intermediate occupations and executives, the main actors with a positive balance as buyers in stable markets.
- In the early 2000, average suburban profiles (*cluster 3*) and stable exurban markets (*cluster 2*, 32% of the cases) transitioned, following two individual trajectories. First, many transitioned from type 3 to type 2 in the early 2000s, or during the pre-crisis years in 2006-07: most of these neighborhoods progressively stabilized after 2007 (*sequence type 3*). Second, their dynamics were characterized by transitions towards *cluster 5* (mature middle-class suburbs) and type 7 (intermediary markets) in *sequence type 4*, in what can be described as dynamic markets. The majority of transitions occurred at two periods of time: between 1999 and 2003; and after 2009. As described by the overall entropy index between 2005 and 2009, these neighborhoods stabilized with a much lower probability of transitioning (Fig 10).
- On their own course, the transitions depicted in sequence type 5 (n = 656, 8.5%) show the highest level of entropy. Starting with a diversity of characteristics, e.g. intermediary markets (*cluster 7*), workers refuges (*cluster 8*), very dynamic mixed markets (*cluster 11*), volatile inflation dynamics clearly fueled a general transition toward dynamic mixed inner suburban markets (*cluster 6*), characterized by a net inflow of intermediate occupations and to some extent independent workers and craftsmen.
- Finally, in *sequence type 6* (n = 359, 4.5%) neighborhoods already in the upper segments in the inner suburbs showed, unsurprisingly, some very stable profiles, and were characterized by the lower proportion of retiree home sales, and a higher proportion of purchases by professionals and executives. They were likely to transition to more exclusive “golden suburban ghettos” (very exclusive wealthy enclaves described as gated, protected and highly desirable spaces of affluence [142]) especially between 1999 and 2003, with growth exclusively fed by the influx of executives and professionals. They occasionally transitioned downward, coming to more stable profiles—type 3, in green in 2009 –.

### Unequal spatial contexts of housing wealth accumulation

These results and successive typologies allows us to better map unequal patterns of pricing, price dynamics, and neighborhood sequences. But prices not only indicate a market value, but are also instrumental in wealth accumulation, between spatial contexts in which price are likely to go up where households are likely to capitalize on assets, and neighborhoods in which price are likely to go down, riskier in terms of investment. In short, lower prices mean a gain in affordability for the lower middle class; but in terms of wealth accumulation, there are winners and loosers. And the probability to pertain to one group or another correlates with the social segregation patterns in the region.

**Fig 12:**
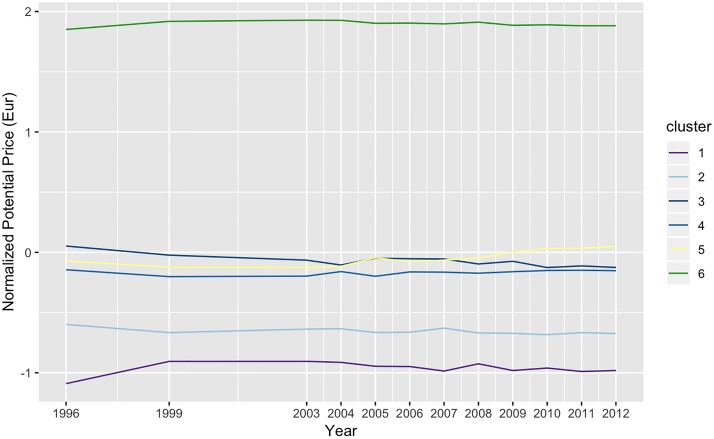
Normalized prices 1996-2012 by neighborhood sequences clusters. Source: BIEN Database, Chambre des Notaires IDF, 2012.

We found a strong support for hypothesis #2: the local trajectories of price appreciation and depreciation correlate with the successive sequences of neighborhoods. A *χ*^2^ analysis confirms that the hypothesis of independence should be strongly rejected (with a *p* − *value* < 2.2*e*^−16^). This analysis shows interesting and contrasted evidences (Fig 12). The normalized graphs shows price trend compared to the annual average: *sequence type 1* as well as *sequence type 2*, areas with a constant influx of retired persons and an average positive balance of intermediate occupations (yellow and light green colors on Fig 2) followed a clearly depreciative trend, compared to the average values: the lower the values, the less likely price went up compared to the rest of the market. Examples on Fig 9 are found in the furthest suburbs of Freneuse, North-West of the region; Montolivet, to the East. For the households owning a property in such contexts and staying in this property, this means less wealth accumulation in real estate, and also the risk of a depreciation of values over time: data clearly show that after 2007-08, values tended to slightly decrease, in a context of a relatively atone market. By contrast, neighborhoods that followed *sequence type 6* in the upper market segments (blue on Fig 2) concentrated the highest values, and prices have remained at the highest level from 1999 to 2012: the highest the value, the highest the probability to protect the investment over time, the highest the probability to accumulate assets, an analysis that has been well framed in how residential markets are structured and protected in suburbia [67]. Many neighborhoods belong to the category in locations such as Feucherolles, Saint-Germain-en Laye, but also Meaux, Coupvray, and large parts of the new towns of Marne la Valle, Fontainebleau, Montereau-Fault-Yonne.

In the mid-market segments, distinct patterns are less clear, because prices belonged to very similar brackets. Data on Fig 12 show however two neighborhood sequences with clearly negative trends. *Sequence type 3* characterized transitions towards mature middle-class suburbs: prices used to be above average in 1996, then went down, with a strong depreciative trend after the crisis in 2007 and 2009. Prices in *sequence type 4* (intermediary markets) also did not appreciate as fast as other contexts, and show stable relative trends. On Fig 9, such neighborhoods are found near Goussainville, Dammartin-en-Goelle, Dourdan, Les-Essarts-le-Roi.

By contrast, owners in *sequence type 5*, dynamic mixed markets with a net inflow of executives, salaried employees and intermediate occupations. Prices increased, affordability decreased, but owners staying in place or selling have been more likely to have accumulated assets over time from 2009 to 2012: in such neighborhoods, prices went up after the global financial crisis. Such results bring a clear depiction and characterization of dynamic middle-class inner suburbs with potential for above-average asset capitalization, supporting hypotheses #2, in contexts that correspond also to suburban gentrification (a net outflow of blue collars (workers). Argentueil, Lagny-sur-Marne, Yerres, Bretigny-sur-Orge, Massy, Pontault-Combault are examples of such a general trend.

To interpret such trends, it shall be reminded that policies have been implemented to stabilize the markets in the aftereffects of the financial crisis on real-estate markets. Many households and real-estate agents were in 2008 expecting the effects of the crisis in France, before the Sarkozy administration took action to aggressively subsidize residential markets with fiscal incentives policies for middle-class and upper-middle-class first-time buyers.

## Conclusion

The main goals of the study were to attempt an analysis of social change with individual-level data, in a property owners market, not relying on census data but on inward and outward flows of sellers and buyers). Instead of explaining prices, we advocate for a spatial approach to model (using interpolation and travel-time matrix), visualize and explain the distribution and dynamics of prices, transactions across the urban fabric. The study provides an analysis of the high level of variability and discrete characteristics of suburban property markets that can hardly by described by over-simplified categories and narratives on suburbanism and spatial relegation of the middle-class, as often found in the French language literature [79, 80].

The main results allows us to get a better understanding of the shapes and spatial dynamics of inequalities in suburbs in the region of Paris. We document the spatial patterns of professionalization (a rise of executives, intermediate occupation and employees) to describe the main trends of inward mobility in property ownership in suburbs (Hypothesis #1), offsetting the outward mobility of retired persons, as sellers, and also the decline of salaried employees and blue collar workers in transactions, except in highly specialized suburbs, the last refuges of the working class in a desindustrialized metropolitan region. In a context of price inflation driven by housing finance regimes and the expansion of credit, we do so by explicitly linking the diverging patterns of appreciation, between local contexts of accumulation with a steady growth of residential prices, and suburbs with declining trends, especially after 2007. In a striking affordability crisis between 1996 and 2012, some suburbs were more likely to attract households building up wealth accumulation through residential real-estate and home values, whereas others became risky contexts with declining values, and for indebted households, negative equity. Volatility of prices and vulnerability of households has progressively clustered in the lower brackets of prices. While increased values and accumulation in well-off neighborhoods of the west-side were expected, we also characterized patterns of price, lower affordability, and higher potential asset-accumulation in the inner suburban middle-class, a lesser known dimension (Hypothesis #2). We finally contextualized the study in order to inform how the maturity of suburbanization in the metropolitan region yields a diversified structure of segregation between the different social classes during the last two decades, that cannot simply oppose rich vs. popular suburbs (Hypothesis #3). It is clear from the different typologies that the maturation of suburbanism has played a role in structuring the stabilization of a vast majority of suburbs and exurbs, while many suburbs also show very active dynamic markets fueled by price inflation and households asset capitalization, not only in the upper-segment, but also in the medium segments.

This analysis forms the basis of a new research agenda, not only in France (ANR WIsDHoM 2019-2022 project “Wealth inequalities and the dynamics of housing market. Interpreting real-estate market-based regime of spatial inequalities”) but more generally in European cities (2018-19 ESPON exploratory program “Big Data for Territorial Analysis and Housing Dynamics”). Because of residential price inflation during the last decade and after the 2007-08 housing and financial markets crisis, it is estimated that 7% of the EU 28 population faced housing costs that accounted for more than half their disposable income. To document both the affordability crisis and unequal patterns of residential wealth and asset accumulation, we deem relevant to measure the financial effort (including data on income and assets), its spatial structure and its distribution among social categories. In this context, it is critical to geographically analyze this ordinary financialization [143] of the “actually existing” real-estate markets [144, 145] for tentative buyers and homeowners: i.e we will spatially analyze wealth dependency of households to market fluctuations, and the increasing necessity for homeowers to behave as asset managers due to the role of housing wealth to secure life trajectories [25]. Using transaction-based spatial datasets, the diversity of trajectories on markets at local levels have been described since the 1990s. Inequalities in terms of financial effort (price vs. income) have increased, while lower-income households have not been excluded from the homeownership market, but displaced in suburbs or rural areas. In further research, using the grid system (conform with the INSPIRE specifications) will allow comparison between the results and methods hereby discussed, and other public data that will progressively be released for this geographical level (e.g. population, income, age…). In order to characterize the local conditions of housing market, we will map the spatial dynamics of the financial effort of households and the unequal dynamics of local affordability, using similar techniques and gridded real-estate data, to harmonize different heterogeneous datasets across cities in Europe.

## Supporting information

S1 Fig Density plot of house values, in Euros, for each categories of buyers 1996-2012. Violin plots represent kernel density estimates. Thresholds defined as 1st decile, first quartile, median, third quartiles and 9th decile; price scale, *log*_10_. Author: R. Le Goix, 2018, UMR Géographie-cités, Labex Dynamite.
(PDF)

S2 Fig Pearson correlation matrices (heatmaps) for percent sellers and buyers in 1996, 2003 and 2012. Author: R. Le Goix, 2018, UMR Géographie-cités, Labex Dynamite.
(PDF)

S3 Fig Potential price (EUR) of single family homes, within 10 min. neighborhoods (animated). Source: BIEN Database, Chambre des Notaires IDF, 2012. Interpolation with R *SpatialPosition R* package, Powerlaw *β* = −0.27, span = 10. Author: R. Le Goix, 2017, UMR Géographie-cités, Labex Dynamite.
(GIF)

S4 Fig Typology of sellers and buyers balance, in single family homes in Ile-de-France, 1996-2012 (animated). Selected years. Source: BIEN Database, Chambre des Notaires IDF, 2012. HCA, euclidian distance, Ward method, N = 7888 cells * 12 years, solution with 11 clusters, *r*^2^ = 61%. Author: R. Le Goix, 2017, UMR Géographie-cités, Labex Dynamite.
(GIF)

S1 Methodological Appendix RMarkdown file, commented code. This file contains a methodological appendix, with full code used to prepare the paper, and datasource identification.
(HTML)

S1 File Zip file containing datasets. This file contains three series of files: gridded data for interpolated property price (S1_File_potential_price_1kgrid_1996_2012.csv), gridded interporlated seller-buyer balance dataset(S1_File_seller_buyer_balance_1kgrid_1996_2012.csv), and a projected RGF93 spatial data file (.SHP) of grid centroids (carroyage_1km_IDF_centroids_RGF93.shp).
(ZIP)
