# Supplementary figures and images for: Who sells to whom in the suburbs? Home price inflation and the dynamics of sellers and buyers in the metropolitan region of Paris, 1996–2012
Source: PLoS One. 2019 Mar 21;14(3):e0213169. doi: 10.1371/journal.pone.0213169 (PMC6428303; doi:10.1371/journal.pone.0213169)

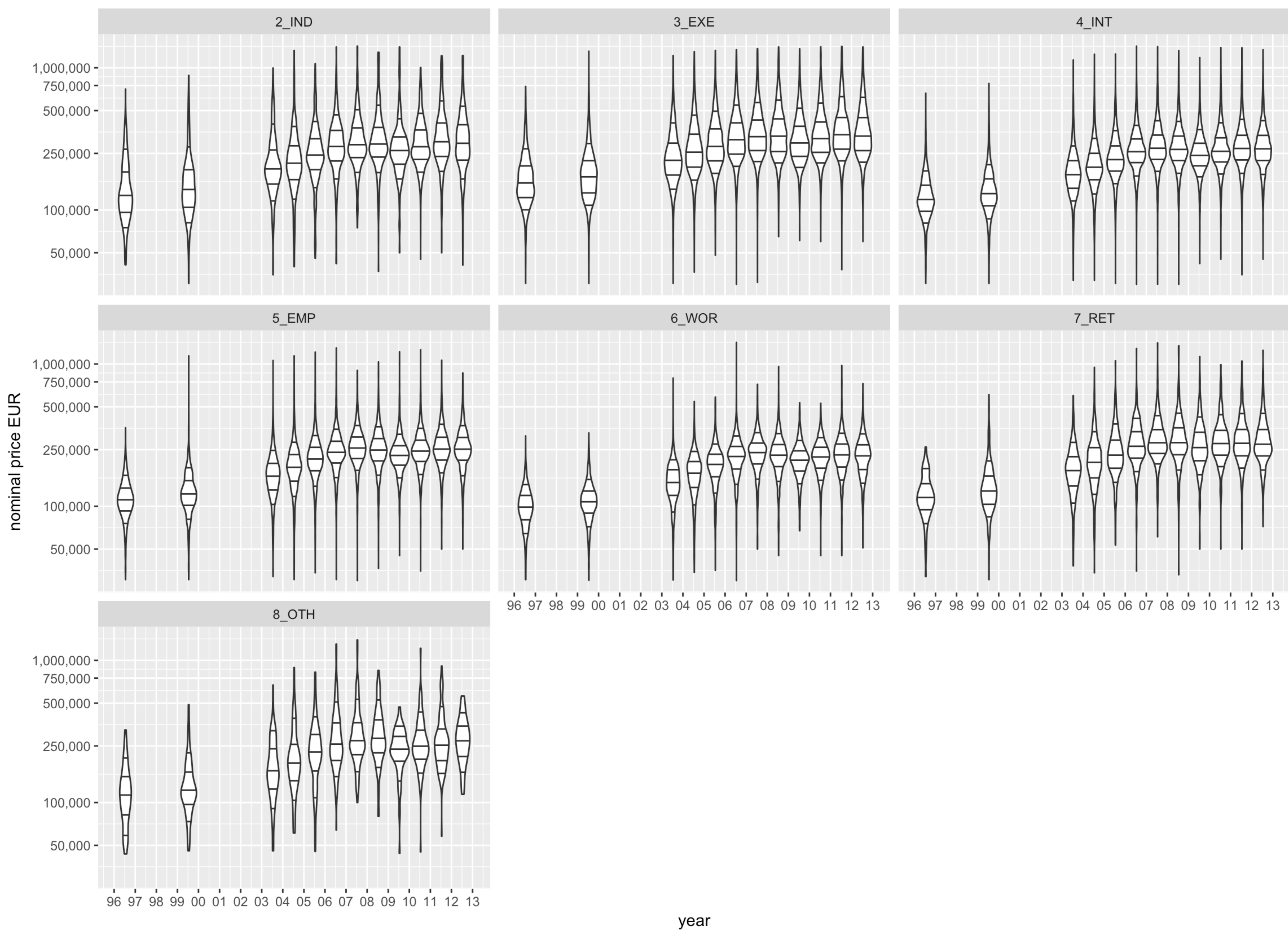

Supplement: S1 Fig — Violin plots represent kernel density estimates. Thresholds defined as 1st decile, first quartile, median, third quartiles and 9th decile; price scale, log10. Author: R. Le Goix, 2018, UMR Géographie-cités, Labex Dynamite. (PDF) [file pone.0213169.s001.pdf]

1996 – Correlation plot

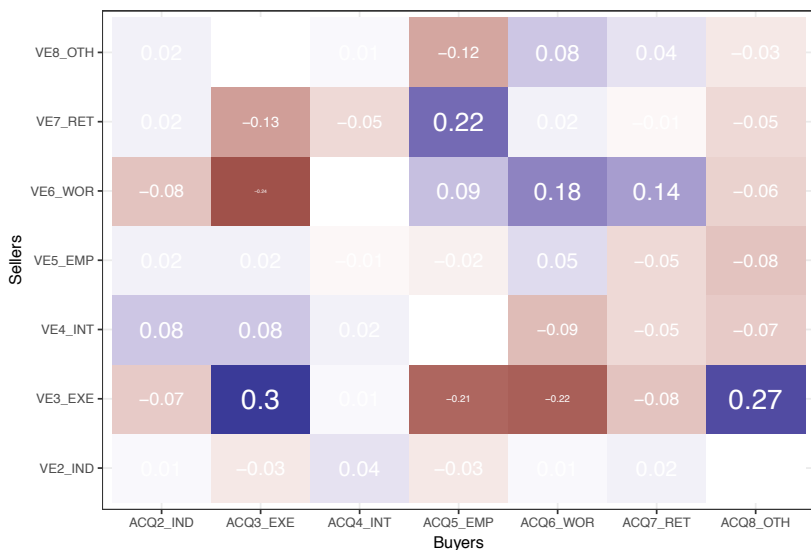

2003 – Correlation plot

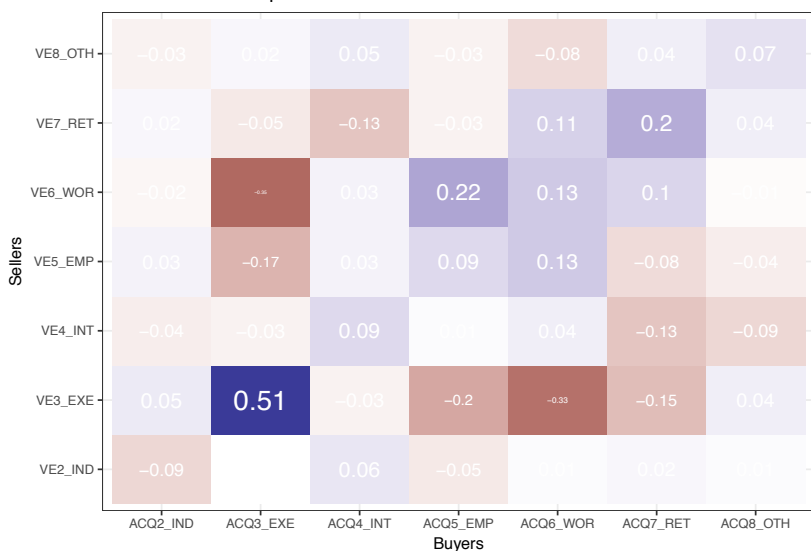

2012 – Correlation plot

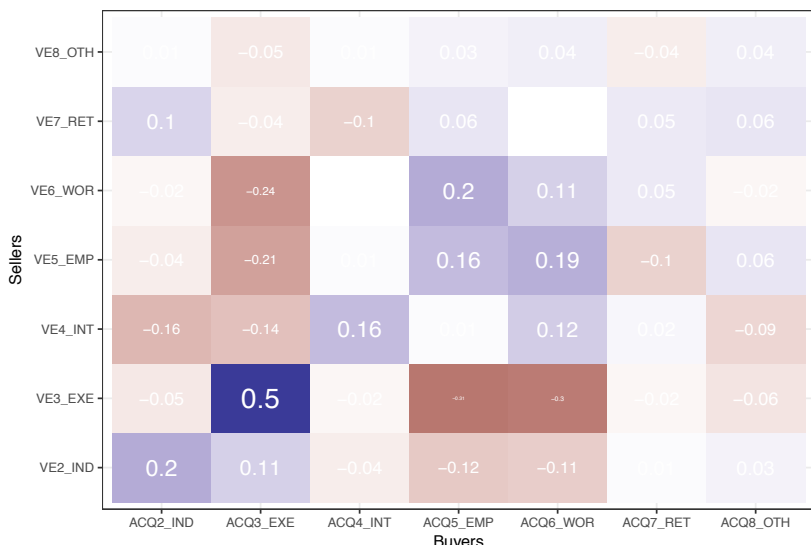

Supplement: S2 Fig — Author: R. Le Goix, 2018, UMR Géographie-cités, Labex Dynamite. (PDF) [file pone.0213169.s002.pdf]

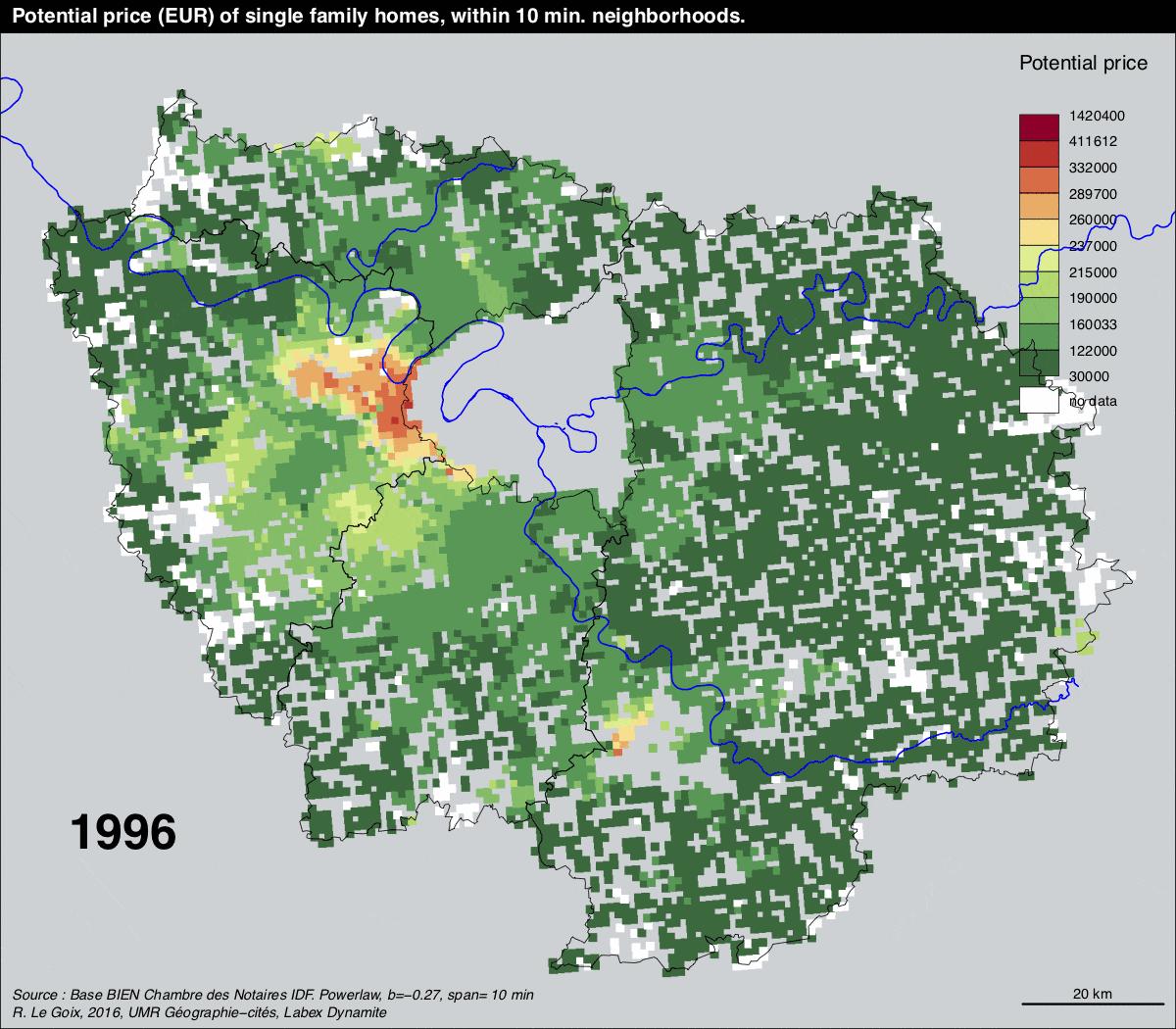

Supplement: S3 Fig — Source: BIEN Database, Chambre des Notaires IDF, 2012. Interpolation with R SpatialPosition R package, Powerlaw β = −0.27, span = 10. Author: R. Le Goix, 2017, UMR Géographie-cités, Labex Dynamite. (GIF) [file pone.0213169.s003.gif]

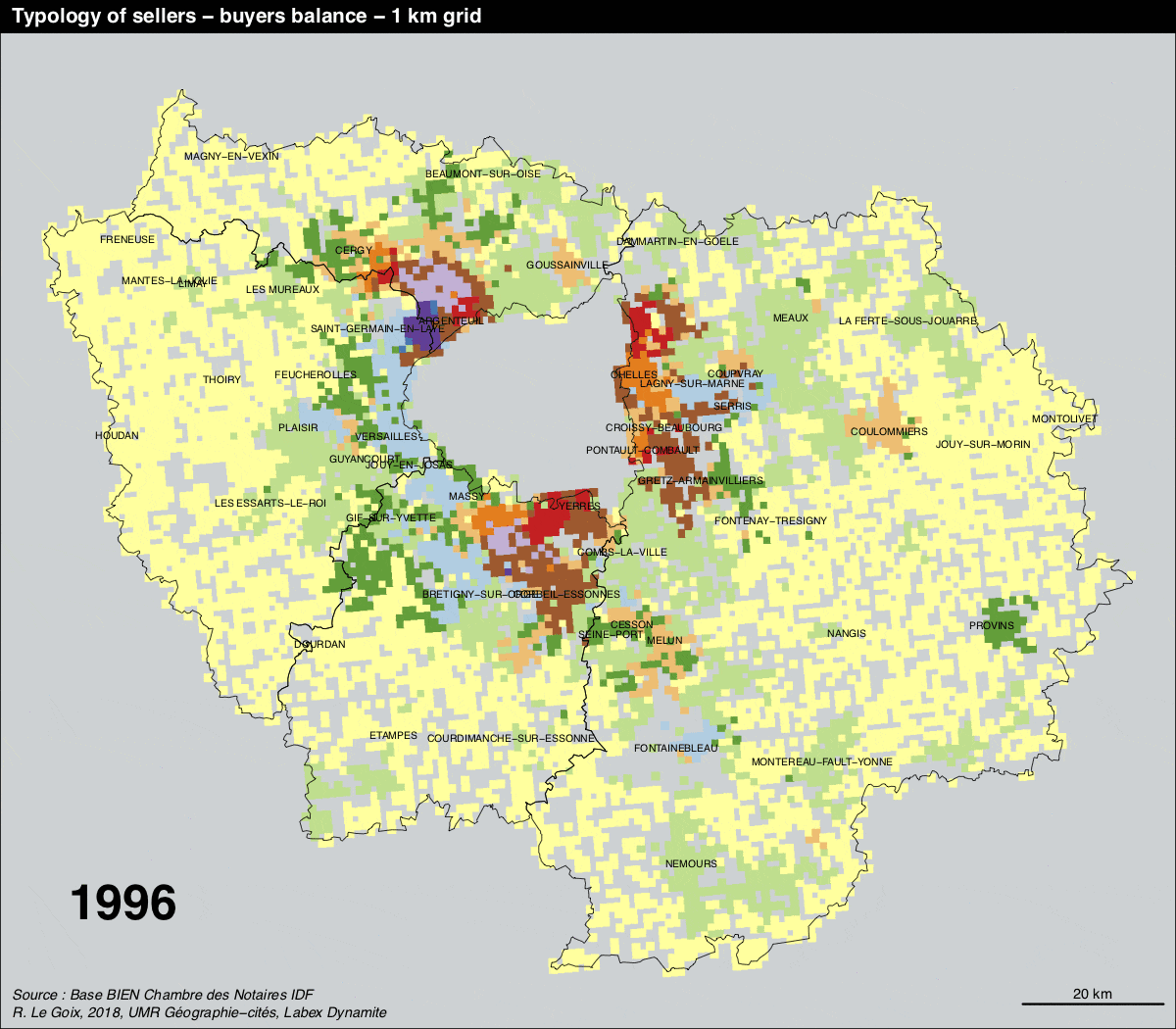

Supplement: S4 Fig — Selected years. Source: BIEN Database, Chambre des Notaires IDF, 2012. HCA, euclidian distance, Ward method, N = 7888 cells * 12 years, solution with 11 clusters, r2 = 61%. Author: R. Le Goix, 2017, UMR Géographie-cités, Labex Dynamite. (GIF) [file pone.0213169.s004.gif]
