# Supplementary material for: Who sells to whom in the suburbs? Home price inflation and the dynamics of sellers and buyers in the metropolitan region of Paris, 1996–2012
Source: PLoS One. 2019 Mar 21;14(3):e0213169. doi: 10.1371/journal.pone.0213169 (PMC6428303; doi:10.1371/journal.pone.0213169)
Supplement: S1 Methodological Appendix — This file contains a methodological appendix, with full code used to prepare the paper, and datasource identification. (HTML) [file pone.0213169.s005.html]

S5 methodological appendix


# S5 methodological appendix

### *’Who sells to whom in the suburbs? Home price inflation and the dynamics of sellers and buyers in the metropolitan region of Paris, 1996-2012*

#### *Renaud Le Goix, Timothée Giraud, Robin Cura, Thibault Le Corre, Julien Migozzi*

#### *12/27/2018*

This commented code (R Markdown document) is published as a methodological appendix of : R. Le Goix, T. Giraud, R. Cura, T. Le Corre, J. Migozzi, (2019) “Who sells to whom in the suburbs? Home price inflation and the dynamics of sellers and buyers in the metropolitan region of Paris, 1996-2012”, *PlosOne*, (first submission, July 2018).

For reference to this document, please use the paper’s citation.

# Load Packages

```
library('rgdal')
# install.packages("SpatialPosition", type = "source") ## Repository & install SpatialPostion
library('SpatialPosition') 
library('ggplot2')
library('RColorBrewer')
library('scales') 
library('reshape') 
library('cluster')
library('ade4')
library('FactoClass')
library('cartography')
library('foreign')
```

# 1. Data sources

## Load database for spatially indexed transactions

The transactions are released as a proprietary database by Paris Notaire Service, a commercial service, on the behalf of the Chamber of the Notaries, under an agreement contracted by the LabEx DynamiTe (ANR-11-LABX-0046) and the Univ. Paris 1 Pantheon-Sorbonne. Paris Notaire Service is a commercial data provider, and does not allow to share commercial data publicly. Datasets used for this study can be obtained through the provider for a fee (for details on accessing and acquiring transactions data: https://basebien.com/PNSPublic/front/f\_basebien.php?rub=1)

*Description of main variables used in the database:*

- `REQ_PRIX` : transactions price paid (institutional data).
- `usage` : main usage of property (residential, commercial…)
- `annee` : year of transaction
- `CSP_AC` : Buyer’s occupational category
- `CSP_VE` : Seller’s occupational category
- `BIDEPT` : *Départment* (district)

```
### Source : Base BIEN, Paris Notaire Service, 2014. Labex Dynamite - UMR Géographie-cités ####
### this database must be resampled for municipalities with more than 10 000 inhabitants
### https://basebien.com/PNSPublic/front/f_basebien.php?rub=1

#Import CSV
BIEN_LABEX <- read.csv("~/SIG/BIEN_Labex/BIEN_LABEX_2016_consolidated_all_years.txt", stringsAsFactors=FALSE)
#968695 observations

dfdata <- BIEN_LABEX

# No missing coordinates and no NA values
dfdata$X <- as.numeric(dfdata$X)
dfdata$Y <- as.numeric(dfdata$Y)
dfdata <- subset(dfdata, X!=0 | Y!=0)
dfdata <- subset(dfdata, X!=1 | Y!=1)
dfdata <- dfdata [!is.na(dfdata$X) ,]
dfdata <- dfdata [!is.na(dfdata$Y) ,]

# Geographical selection:  to the outer suburban districts
dfdata <- subset(dfdata, BIDEPT=="77" | BIDEPT=="78"|BIDEPT=="91"|BIDEPT=="95")

# ordinary transactions (OTC) btw sellers and buyers "gré à gré" ; residential only ;  price >= 1 eur
dfdata <- subset(dfdata, REQ_MUT==1 && USAGE=="HA" && REQ_PRIX >= 1 )

#Houses only  
dfdata <- subset(dfdata, REQTYPBIEN=="M" | REQTYPBIEN=="MA")
# Single family only (pavillon)
dfdata <- subset(dfdata, TYPMAI=="PV" | TYPMAI=="VI")


g <- ggplot(dfdata) +
  geom_histogram (aes(x = annee),
                  stat = "bin") + 
  facet_wrap(~ BIDEPT) + 
   labs(title ="Count of transactions by district (département)", x = "year", y = "count") +
  theme_bw()

g

# 159251 observations
```

## Load grid centroids and spatial boundary files.

Grid centroids and spatial boundary files are IGN and INSEE public data that can be downloaded from the following sources :

- downloadSpatial data from IGN : http://professionnels.ign.fr/enseignement-recherche
- Grid data files : https://www.insee.fr/fr/statistiques/1405815.

```
#### 1.2. Grid Centroids ####
centroidesCarroyage <- readOGR(dsn="analyses_carroyage_1km_avec_stewart/carroyage_1km_GC_centroids_RGF93.shp",
                               layer = "carroyage_1km_GC_centroids_RGF93",
                               encoding="utf8", stringsAsFactors=FALSE)


# Temp grid files for results
carroyage <- centroidesCarroyage

#### 1.3 Other shapefiles for cartography purpose #####

departements <- readOGR(dsn = "analyses_carroyage_1km_avec_stewart/departements_GC_merged_RGF93.shp",
                        layer = "departements_GC_merged_RGF93", 
                        encoding = "utf8", stringsAsFactors = FALSE)

departements_carto <- readOGR(dsn = "analyses_carroyage_1km_avec_stewart/departements_GC_RGF93.shp",
                              layer = "departements_GC_RGF93", 
                              encoding = "utf8", stringsAsFactors = FALSE)

CartoMask <- readOGR(dsn = "analyses_car1km_apt_avec_stewart/departements_RGF93.shp",
                     layer = "departements_RGF93", 
                     encoding = "utf8", stringsAsFactors = FALSE)

Fleuves <- readOGR(dsn = "fleuves_rivieres_main.shp",
                   layer = "fleuves_rivieres_main", 
                   encoding = "utf8", stringsAsFactors = FALSE)

labels <- readOGR(dsn = "analyses_carroyage_1km_avec_stewart/Selection_com_for_labels.shp",
                  layer = "Selection_com_for_labels", 
                  encoding = "utf8", stringsAsFactors = FALSE)
```

## Resampling

The sample of transactions acquired is composed of all transactions for municipalities of 10,000 inhabitants or less in 2011, and a random 50% sampling of transactions for all larger municipalities. This sample was finally adjusted: using the property price, the first and last percentile of transactions was withdrawn from the sample because of inaccuracies in some price data coding, resulting in very low or unreliably high prices for single family homes. Furthermore, to offset the imbalance in the frequencies of transactions in municipalities of more than 10,000 inhabitants, the random sample of transaction was duplicated in such cases, resulting in a correct total number of weights in the redressed sample of 206,126 transactions. Redressing the total weight of transactions did not affect the statistical distributions of values, and was a necessary procedure to perform the subsequent data processing (Fig 3).

```
##### 1.3 Resample database for municipalities > 10 000 hab ######
### Read municipalities DB ###
communes <- read.dbf("communes_pop2011.dbf")
communes$weight <-ifelse (communes$POP2011 > 10000,2,1) # weight is 2 if pop > 10000 

# #Merge municipalities and dfdata
# 'insee' = municipality ID
communes$POP2011 <- NULL
colnames(communes)[1] <- "insee"
communes$INSEE <- NULL
communes$insee <- as.character(communes$insee)
dfdata <-  merge(dfdata, communes, by = "insee", all.x=TRUE)
```

## Matching data to a census grid using a travel-time matrix

This grid used combines three main advantages for a study of suburban areas - A 1 km-cell can be considered as an appropriate proxy for homogeneous areas matching the fragmented suburban built environment. Although suburban municipalities are often small in size, an average of 820 inhabitants (Charmes 2009), several subdivisions, with different characteristics of housing, are generally included within municipal boundaries (Fig 4). As discussed in the literature review, visualizations and analysis are generally conducted at the municipal level, whereas the level of heterogeneity and submarkets would probably be best defined at the level of the subdivision, given that the combination of secondary street segments generally define local submarkets (Xiao, Webster, and Orford 2016, 1506, 1781).

- Because of protection of individual data and statistical secrecy, only cells for 11 inhabitants or more have been constructed into the spatial layer: by using it so, the analysis does not impute a value to areas that have no values, or no potential buyers or sellers. As a manner of clarification, many national forests, golf courses and other immutable land uses, large heritage landmarks (Fontainebleau, Versailles, Vaux-le-Vicomte…) have no population and therefore no potential housing value, but are also considered as valuable locational amenities that yield strong positive externalities for the valuation of homes.
- Matching the “real estate agent paradigm”, **i.e.** assessing the price of a property with reference to nearby similar properties, the distance matrix for the interpolation uses a travel-time matrix between cell centroids: the potential values will be therefore imputed to the closest cells, making two nearby properties more likely to be priced equally (spatial interaction hypothesis) if cells are connected by local streets. As on Fig 4, with a radius of 10 min, spatial interactions for Ozoir-la-Ferrière will more likely occur with Roissy-en-Brie, while the barrier effect will not allow interactions with property values on the east side of Ozoir. In other words, a travel-time matrix better renders the barrier effects existing between neighborhoods and in municipalities, in fragmented built environments made of green belt and buffer zones between neighborhoods.

### Create distance matrix

```
### Create distance matrix using OSRM server
### Run this cunk of code only once

library(rgdal)
library(osrm)
options(osrm.server = "http://0.0.0.0:5000/")
com <- readOGR(dsn = "data/", layer = "carroyage")

plot(com)
row.names(com) <- com$ID
t0 <- Sys.time()
com_table <- osrmTable(loc = com)
t1 <- Sys.time()
round(t1 - t0,1)


save(list = c('com_table', 'com'), file = "DistMatIDF/data/distances.RData")
```

### Load distance matrix

```
##### 1.3.2. Load travel-time matrix ####
load("DistMatIDF/data/distances.RData")
DistMatrix <- com_table$durations
```

### Match transactions and grid

```
##### 1.5 Merge db with carroyage ######
### Export ID biens et coord pour GIS ###

# Extract transaction data for spatial join avec 'Carroyages'. Run only once
temp <- dfdata[,c("ID", "X", "Y")]
write.csv(temp,"bien_pour_carroyage_1km.csv")


### Transaction file has been previously controlled and prepared in a GIS (QGIS), with a spatial joint (points included within each cell), creating a field 'id_carreau' with a grid cell spatial ID.

Biens_Carroyage <- read.dbf("temp_pour_matchBIENconsolidee_carroyage1km/Biens_carroyage1km_avecID_carreaux.dbf")
Biens_Carroyage <- Biens_Carroyage[c("ID","ID_1")]
### Then merge
names(Biens_Carroyage)[1] <- "ID"
Biens_Carroyage$id_carreau <- NULL
names(Biens_Carroyage)[2] <- "id_carreau"
dfdata <-  merge(dfdata, Biens_Carroyage, by = "ID")
# knownpoints <- merge(centroidesCarroyage, Carreau_sum, by = "id")

# If no matching cell, then drop
dfdata <- dfdata [!is.na(dfdata$id_carreau) ,]
dfdata <- dfdata [!is.na(dfdata$weight) ,]
dfdata <- dfdata[!(dfdata$id_carreau==""),] #Remove empty rows
# 152449 obs remaining, matching with grid cells
sum(dfdata$weight)
# and 206126 weights
```

### Recode socioprofessional categories

Original files contain a detailled typology of socio-economic profiles of occupations for buyers and sellers. For harmonization purposes and for the sake of the clarification when interpreting results, groups are merged together, using the standard 1 digit INSEE typology.

```
#### 1.6 Prepare dataset for seller-buyers analysis ####

#Load CS socio longs traduits
CS_Cat_Fr_EN <- read.csv("~/SIG/BIEN_Labex/analyses_carroyage_1km_avec_stewart/CS_Cat_Fr_EN_short.csv", sep=";")


require (RcmdrMisc) # For recode function
dfdata$CSP_AC_long <- dfdata$CSP_AC
dfdata <- within(dfdata, {
  CSP_AC <- Recode(CSP_AC_long, '1:19 = "1_FAR"; 2:29 = "2_IND"; 3:39 = "3_EXE"; 4:49 = "4_INT"; 
                           5:59 = "5_EMP"; 60 = "6_WOR"; 70 = "7_RET"; 80 = "8_OTH" ; 90 = "9_REP" ; "" = NA')
})

dfdata$CSP_VE_long <- dfdata$CSP_VE
dfdata <- within(dfdata, {
  CSP_VE <- Recode(CSP_VE_long, '1:19 = "1_FAR"; 2:29 = "2_IND"; 3:39 = "3_EXE"; 4:49 = "4_INT"; 
                           5:59 = "5_EMP"; 60 = "6_WOR"; 70 = "7_RET"; 80 = "8_OTH" ; 90 = "9_REP" ; "" = NA')
})

detach("package:RcmdrMisc", unload=TRUE)

dfdata$CSP_VE <- as.factor(dfdata$CSP_VE)
dfdata$CSP_AC <- as.factor(dfdata$CSP_AC)

head(dfdata)
```

# 2. Interpolation of discrete socioeconomic data applied to price

The methodology also relies on an interpolation of discrete socioeconomic phenomena: prices and sellers / buyers occupational categories. We propose an approach that computes a synthetic value based on distance and weight of the observed population, as initially proposed by Stewart (Stewart 1942) for an analysis of the distribution of student population and catchment areas of American Universities, and more recently applied for socioeconomic phenomena (Grasland 2009).

## Details on methodology

Stewart’s potential can be considered as similar to spatial interpolation method such as IDW (Inverse Distance Weighting) and kernel density estimator, estimating unknown values of non-observed points from known values given for every known measured values in a given location. But Stewart’s model should more be considered as a spatial interaction model (Commenges and Giraud 2016). For social interactions, the most common formulation is the gravity model, that Tobler and Wineburg (Tobler and Wineburg 1971) generalized to many social facts: “distance may be in hours, dollars, or kilometers; populations may be in income, numbers of people, numbers of telephones and so on; and the interaction may be in numbers of letters exchanged, number of marriages, similarity of artifacts or cultural traits and so on” (Tobler and Wineburg 1971). As in many fields in human geography, spatial interactions between house price, and variables describing the market structure, derive from a function of distance between \(i\) and \(j\), a weight variable (e.g. population) \(P\_{i}\) and \(P\_{j}\), and a constant depending of the phenomena.

From this, we infer that property markets are discrete social data, similar to Tobler’s hypothesis: a potential price for a specific location is a function of distance to nearby similar transactions, and also a function of the number of properties available, turnover and realized transactions.

We can think of this problem by analogy, considering that market actors (i.e. real-estate agent, sellers and buyers) need to gather information on nearby transactions and homes to accurately price the advertised property, to efficiently negotiate pricing, to publicize the listed property, and for the sellers and buyers to engage in residential mobility, therefore expanding the concept of intervening opportunities (Stouffer 1940) to a wide series of socio-spatial interactions on real-estate markets. This can be generalized into a more formalized hypothesis : the interactions between property values, between sellers and buyers, are a function of distance and a function of the number of properties and individuals (sellers / buyers) on the market.

## Compute density of transactions and potential price

```
#################################
##### Univariate analysis  ######
#################################


# Compute quantiles for all years -> harmonizing thresholds for cartography
# Exclude first and last 0,1% to avoid outliers, and unreliable values
dfdata$REQ_PRIX <- as.numeric(dfdata$REQ_PRIX)
bornesQuantiles_prix <- quantile(dfdata$REQ_PRIX, 
                                 probs = seq(0,1, 0.001), 
                                 na.rm=TRUE)
bornesQuantiles_prix

# Cleanup : remove the first and last (lower and highest) 0,01% transactions
dfdata <- subset(dfdata, REQ_PRIX >= 29951 & REQ_PRIX <= 1421463)

# Prepare decile threshold for cartography
bornesQuantiles_prix <- quantile(dfdata$REQ_PRIX, 
                                 probs = seq(0,1, 0.1), #Quantiles en déciles
                                 na.rm=TRUE)
bornesQuantiles_prix

#### 2.1.2. Boxplot  #####
# boxplot by years and subsequent univariate and bivariate analysis
pavillons <- dfdata[, c("annee","REQ_PRIX", "CSP_AC", "CSP_VE")]
pavillons <- within(pavillons, {annee <- as.factor(annee)})
library(lubridate)
pavillons$annee <- as.Date(as.character(pavillons$annee), format = "%Y")

## Log
g <- ggplot(aes(y = REQ_PRIX, x = annee, group = annee), data = pavillons) +
  geom_boxplot() +
  scale_x_date(labels = date_format("%y"), breaks = date_breaks("1 years")) + 
  scale_y_log10(breaks=c(50000,100000,250000,500000,750000,1000000),labels=c("50,000","100,000","250,000","500,000","750,000","1,000,000"))+
  annotation_logticks()  +
  xlab('year') +
  ylab('nominal price EUR (log scale)')
g

## Normale
g <- ggplot(aes(y = REQ_PRIX, x = annee, group = annee), data = pavillons) +
  geom_boxplot() +
  scale_x_date(labels = date_format("%y"), breaks = date_breaks("1 years")) + 
  scale_y_continuous(breaks=c(50000,100000,250000,500000,750000,1000000,2000000),labels=c("50,000","100,000","250,000","500,000","750,000","1,000,000","2,000,000"))+
  xlab('year') +
  ylab('nominal price EUR')
g


#### 2.1.3. Density violin plots #####
g <- ggplot(aes(y = REQ_PRIX, x = annee, group = annee), data = pavillons) +
  geom_violin(draw_quantiles = c(0.10, 0.25, 0.5, 0.75, 0.9)) +
  scale_x_date(labels = date_format("%y"), breaks = date_breaks("1 years")) + 
  scale_y_log10(breaks=c(50000,100000,250000,500000,750000,1000000),labels=c("50,000","100,000","250,000","500,000","750,000","1,000,000"))+
  xlab('year') +
  ylab('nominal price EUR')
g


#### 2.1.2. Density violin plots by buyers #####
####nb  no farmers, no NA ####
sub_pavillons <- subset(pavillons, CSP_AC!="NA" & CSP_AC!="1_FAR")

g <- ggplot(aes(y = REQ_PRIX, x = annee, group = annee), data = sub_pavillons) +
  facet_wrap(~ CSP_AC) +
  geom_violin(draw_quantiles = c(0.10, 0.25, 0.5, 0.75, 0.9)) +
  scale_x_date(labels = date_format("%y"), breaks = date_breaks("1 years")) + 
  scale_y_log10(breaks=c(50000,100000,250000,500000,750000,1000000),labels=c("50,000","100,000","250,000","500,000","750,000","1,000,000"))+
  xlab('year') +
  ylab('nominal price EUR')
g


###############################
##### General operations ######
###############################

##### Compute densities of transactions for each cell
Carreau_sum_all <- aggregate(dfdata[, c("weight")],
                             by = list(dfdata$id_carreau),
                             sum)
Carreau_sum_all$densite <- Carreau_sum_all$x   # grid cells = 1 sq.km
bornesQuantiles_densite <- quantile(Carreau_sum_all$densite, 
                                    probs = seq(0,1, 0.1),
                                    na.rm=TRUE)

bornesQuantiles_densite
rm (Carreau_sum_all)


bornesQuantiles_prix
bornesQuantiles_densite

#Set color palette
colPal <- rev(brewer.pal(n = 10, name = "RdYlGn"))

# Create a vector containing the list of years to be analyzed
mesAnnees <- unique(dfdata$annee) 
mesAnnees <- sort(mesAnnees)

# Following lines are used for testing purpose, to run the code for a subset of years
#mesAnnees <- c(1996,2012)
#currentYear <- 2005
```

### Estimation of the powerlaw parameter bêta with semi-variograms for spatial interaction models

```
#########################
#### Semi-variograms ####
#########################

## Run this chunk of code once to estimate the parameters for stewart interpolation.

## Load spatial packages

library(maps)         ## Projections
library(maptools)     ## Data management
library(sp)           ## Data management
library(spdep)        ## Spatial autocorrelation
library(gstat)        ## Geostatistics
library(splancs)      ## Kernel Density
library(spatstat)     ## Geostatistics
library(pgirmess)     ## Spatial autocorrelation
library(RColorBrewer) ## Visualization
library(classInt)     ## Class intervals
library(spgwr)        ## GWR
library(lattice)


## recharger les libs normales pour suite analyse
## Faire tourner les variograms à part de l'analyse acq vendeur, en raison de "conflits", entre les packages
## A faire à la main, nécessite un contrôle systématique

currentYear <- 2012
#for (currentYear in mesAnnees){

print(paste(currentYear,  sep = " - "))
# On isole les données
currentData <- dfdata[dfdata$annee == currentYear,]
#   if (nrow(currentData) == 0){
#     # Il n'y a pas de ventes de cette année et de cette CSP,
#     # on ne fait rien
#     print("Pas de transaction pour cette combinaison")
#   } else {
#     #### AGREGATION DONNEES ET CALCUL KNOWNPOINTS
currentData$price <- (currentData$REQ_PRIX * currentData$weight)   #multiplier prix par mettre carré pour poids
Carreau_sum <- aggregate(currentData[, c("price","weight")],
                         by = list(currentData$id_carreau),
                         sum)
Carreau_sum$PRIX_MOY <- Carreau_sum$price / Carreau_sum$weight


### Creation knownpoints
names(Carreau_sum)[1] <- "ID"
Carreau_sum$id <- as.character(Carreau_sum$ID)
spatPts <- merge(centroidesCarroyage, Carreau_sum, by = "ID")


spatPts <- spatPts[!is.na(spatPts@data$weight) ,] #  Remove rows in spatial dataframe

## Take a look at the payload variable

bubble(spatPts,"PRIX_MOY")

dev.off()

## Sample variogram

plot(variogram(log(spatPts@data$PRIX_MOY)~1, data=spatPts, cloud=F),type="b",pch=16)

## Sample variogram
#plot(variogram(log(spatPts@data$PRIX_MOY)~1, locations=coordinates(spatPts), data=spatPts, cloud=T),pch=16, cex=1)


## Identify outlying pairs

v <- variogram(log(spatPts$PRIX_MOY) ~ 1, data=spatPts)
v

print(xyplot(gamma ~ dist, v, ylim=c(0,max(v$gamma)+2*sd(v$gamma)), pch = 3, type = 'b', lwd = 2,
             panel = function(x, y, ...) {
               for (i in 1:50) {
                 spatPts$random <- sample(spatPts$PRIX_MOY)
                 v <- variogram(log(random) ~ 1,  data=spatPts)
                 llines(v$dist, v$gamma, col = 'grey')
               }
               panel.xyplot(x, y, ...)
             },
             xlab = 'distance', ylab = 'semivariance'
))


## Fit variogram with exponential model
v
vgm()
v.fit <- fit.variogram(v, vgm(psill=1, model="Pow", range=1))

v.fit
plot(v, v.fit, pch = 16,cex=.5)

dev.off()


rm(spatPts)

# Results for 1996, 2005 and 2012
#model       psill     range
#1996   Pow 0.01119127 0.2462626
#2005   Pow 0.008231626 0.2610676
#2012   Pow 0.004708944 0.3173345

# average fit = 0.27
```

## Compute the stewart interaction model for price, and generate maps for pontential price 1996-2012

We apply Stewart’s potential to house price, using the `SpatialPosition` R package (Commenges, Giraud, and Boulier 2015, 1999). The potential of population is generally defined as a stock of population weighted by distance, where \(A\_{i}\) the potential of \(i\), \(O\_{i}\) the stock of population at \(j\), \(f(d\_{ij})\) a negative function of distance, generally a power or exponential curve. Function parameters have been estimated by the means of semi-variograms, i.e. an estimation of the spatial variability (the variance of a parameter considering the lag or distance between pairs of datapoints). We have elected to implement a Pareto function, with a span of 10 minutes (travel-time by street network) and a \(\beta\) parameter of 0.27; \(\alpha\) is defined as the distance where the density of probability of the spatial interaction function equals 0.5, as documented in the `SpatialPosition` package.

The computation of the price potential follows a two step procedure : first, the potential for price is computed as the potential total value in a cell (\(Pp\)) ; then, the potential for the number of transactions (\(Pt\)) in a given cell is computed. The potential house price for a given cell equals to \(Pp/Pt\), and then matched to a grid and mapped.

This chunk of code generates the file of gridded potential prices, made available as *Supporting information S6 File*.

```
#########################################
###### Loops stewart interpolation ######
#########################################

row.names(centroidesCarroyage) <- centroidesCarroyage$ID # Create rownames

for (currentYear in mesAnnees){
  
  print(paste(currentYear,  sep = " - "))
  currentData <- dfdata[dfdata$annee == currentYear,]
  if (nrow(currentData) == 0){     
    print("No data for this selection")
  } else {
    #### aggregate data and compute values for knownpoints
    currentData$price <- (currentData$REQ_PRIX * currentData$weight)   #values * weight for each cell.
    Carreau_sum <- aggregate(currentData[, c("price","weight")],
                             by = list(currentData$id_carreau),
                             sum)
 
    #### Create knownpoints
    names(Carreau_sum)[1] <- "ID"
    Carreau_sum$id <- as.character(Carreau_sum$ID)
    knownpoints <- merge(centroidesCarroyage, Carreau_sum, by = "ID")

    #### Compute matrix
    currentStewartPrix <- stewart(knownpts = knownpoints,
                                  unknownpts = centroidesCarroyage,
                                  matdist = DistMatrix, varname = "price",### multiplied by weight
                                  typefct = "pareto", span = 10, 
                                  beta = -0.27, longlat = FALSE)
    currentStewartWeight <- stewart(knownpts = knownpoints,
                                    unknownpts = centroidesCarroyage,
                                    matdist = DistMatrix, varname = "weight",
                                    typefct = "pareto", span = 10,
                                    beta = -0.27, longlat = FALSE)
    if (!identical(currentStewartPrix@data$ID, currentStewartWeight@data$ID)){
      print(sprintf("Error for year %s ", currentYear))
    }
    
    
    # Map of the average value of price potential
    
    currentStewartPrix@data$MOY_PRIX_M <- (currentStewartPrix@data$OUTPUT / currentStewartWeight@data$OUTPUT) #weighted mean : total values by cell / nb of weights
    
    currentStewartPrix@data$nb <- 1 # trick to set the symbol size for cartography 
    currentStewartWeight@data$nb <- 1
 
#    pdf(file=sprintf("analyses_carroyage_1km_avec_stewart/par_annee/maps_by_year/price_annee_%s_EN_nocaptionV3.pdf", currentYear), width=8, height=7,useDingbats=FALSE) # Uncomment to produce a pdf output
     opar <- par(mar = c(0,0,1.1,0)) #If layoutlayer
#    opar <- par(mar = c(0,0,0,0)) #Without layout layer
    plot(CartoMask, add = F, border = "NA", bg = "#cdd2d4")
    
    # Map the regional potential price for any given year
    propSymbolsChoroLayer(spdf = currentStewartPrix, df = currentStewartPrix@data,
                          spdfid = "ID", dfid = "ID",
                          var = "nb", var2="MOY_PRIX_M",
                          breaks = bornesQuantiles_prix, col = colPal, 
                          inches = 0.06, 
                          symbols = "square", lw= NULL, border = NULL,
                          legend.var.pos = "n",
                          legend.var2.pos = "topright",
                          legend.var2.title.txt = "Potential price \n",
                          legend.var2.values.rnd = 0,
                          add=T)
    
    plot(Fleuves, add=T, lwd = 1, border = "blue", col = "blue")
    plot(departements_carto, add=T, lwd = 0.5, border = "black")
    # Set a text to explicit the function parameters
    text(x = 590000, y = 6800000, 
         labels = currentYear, 
         cex = 2, adj = 0, font = 2)
    layoutLayer(title = "Potential price (EUR) of single family homes, within 10 min. neighborhoods.", 
                sources = "Source : Base BIEN Chambre des Notaires IDF. Powerlaw, b=-0.27, span= 10 min", 
                author = "R. Le Goix, 2016, UMR Géographie-cités, Labex Dynamite")    
    
    par(opar)
    dev.off()     
    
    # Map transaction density
    currentStewartWeight@data$densite <- currentStewartWeight@data$OUTPUT
    
#    pdf(file=sprintf("analyses_carroyage_1km_avec_stewart/par_annee/maps_by_year/densite_annee_%s_EN_V3.pdf", currentYear), width=8, height=7,useDingbats=FALSE) # Uncomment to produce a pdf output
    opar <- par(mar = c(0,0,1.1,0))
    plot(CartoMask, add = F, border = "NA", bg = "#cdd2d4")
    
    # Map the regional potential density 
    propSymbolsChoroLayer(spdf = currentStewartWeight, df = currentStewartWeight@data,
                          spdfid = "ID", dfid = "ID",
                          var = "nb", var2="densite",
                          breaks = bornesQuantiles_densite, col = colPal, 
                          inches = 0.06,
                          symbols = "square", lw= NULL, border = NULL,
                          legend.var.pos = "n",
                          legend.var2.pos = "topright",
                          legend.var2.title.txt = "Transactions potential densities",
                          legend.var2.values.rnd = 2,
                          add=T)
    
    plot(Fleuves, add=T, lwd = 1, border = "blue", col = "blue")
    plot(departements_carto, add=T, lwd = 0.5, border = "black")
    # Set a text to explicit the function parameters
    text(x = 590000, y = 6800000, 
         labels = currentYear, 
         cex = 2, adj = 0, font = 2)
    layoutLayer(title = "Density of transactions, single family homes, in 10 min neighborhoods", 
                sources = "Source : Base BIEN Chambre des Notaires IDF. Powerlaw, b=-0.27, span= 10 min", 
                author = "R. Le Goix, 2016, UMR Géographie-cités, Labex Dynamite")
    
    
    par(opar)
    dev.off()     
    
    
    # Compile results 
    currentWeightedPrice <- currentStewartPrix@data$OUTPUT / currentStewartWeight@data$OUTPUT
    currentName <- paste("Prix", currentYear)
    carroyage[[currentName]] <- currentWeightedPrice
    currentName <- paste("Freq", currentYear)
    carroyage[[currentName]] <- currentStewartWeight@data$OUTPUT
    currentName <- paste("Dens", currentYear)
    carroyage[[currentName]] <- currentStewartWeight@data$OUTPUT 
    rm(currentData,  currentStewartPrix, currentStewartWeight)
    
    
    
  }
}

summary(carroyage)

write.csv(carroyage,"S6_potential_price_1kgrid_1996_2012.csv")
```

## Typology of house price dynamics, 1996-2012.

To characterize change and local patterns of inflation, we apply a cluster analysis based on property prices.

```
# Use S6_potential_price_1kgrid_1996_2012.csv to replicate and merge it within 'carroyage@data' spatial data frame


# If working on spatial data frame, uncomment the 2 following lines
selecVar <- c("ID", "Prix 1996","Prix 1999","Prix 2003","Prix 2004","Prix 2005", "Prix 2006", "Prix 2007", "Prix 2008", "Prix 2009","Prix 2010","Prix 2011", "Prix 2012") 
PourCAH <- carroyage@data[, selecVar]

# Set color palette
cahPal <- c( "#6A3D9A", "#A6CEE3", "#045a8d","#1F78B4","#FFFF99",
             "#33A02C", "#FB9A99", "#B2DF8A", "#FF7F00","#E31A1C",
             "#FDBF6F","#CAB2D6")

pie(rep(1, 12), col = cahPal) #Checking palette.

########################################
### HCA  chi2 on prices              ###
### cf. http://rpubs.com/RobinC/AFC_CAH 
### pour CAH chi2 sur les prix       ###
########################################

# #Rownames = GEOID
rownames(PourCAH) <- as.character(PourCAH$ID) 
PourCAH$ID <- NULL
#remove if NA
PourCAH<-PourCAH[complete.cases(PourCAH),]


AFC <- dudi.coa(df=PourCAH, scannf=FALSE, nf=ncol(PourCAH))
plot.dudi(AFC, labels=F)
distMat <- dist.dudi(AFC, amongrow=TRUE)  #Prepares  Chi2 dsitance matrix

CAH <- ward.cluster(distMat, peso = apply(X=PourCAH, MARGIN=1, FUN=sum) , plots = TRUE, h.clust = 1) # Applies HCA on raw prices
## The "ward" method has been renamed to "ward.D"; note new "ward.D2"

#uncomment pdf and dev.off() to generate PDF outputs
#pdf(file="analyses_carroyage_1km_avec_stewart/typo_cah_resultats_R/inertia_bar_plots.pdf",width=6.5,height=5)
par(mfrow=c(2,2))
barplot(sort(CAH$height / sum(CAH$height), decreasing = TRUE)[1:100] * 100,
        xlab = "Nodes", ylab = "% of total inertia",
        names.arg=1:100, main="Inertia for partitions")

barplot(cumsum(sort(CAH$height / sum(CAH$height), decreasing = TRUE))[1:100] * 100,
        xlab = "Nb of clusters k", ylab = "% of total inertia",
        names.arg=1:100, main="Inertia")

barplot(sort(CAH$height / sum(CAH$height), decreasing = TRUE)[1:20] * 100,
        xlab = "Nodes", ylab = "% of total inertia",
        names.arg=1:20, main="Inertia for partitions")

barplot(cumsum(sort(CAH$height / sum(CAH$height), decreasing = TRUE))[1:20] * 100,
        xlab = "Nb of clusters k", ylab = "% of total inertia",
        names.arg=1:20, main="Inertia")
#dev.off()

#
#pdf(file="analyses_carroyage_1km_avec_stewart/typo_cah_resultats_R/dendrogram.pdf",width=6.5,height=5)
par(mfrow=c(1,1))
plot(as.dendrogram(CAH), leaflab = "none")
#dev.off()

# 10 clusters -> 25% of variance
PourCAH$clusters <- cutree(tree = CAH, k = 10) ### Determine where to cut the tree (nb of clusters)
PourCAH$clusnames <- as.factor(PourCAH$clusters)

#pdf(file="analyses_carroyage_1km_avec_stewart/typo_cah_resultats_R/PCA_with_typology.pdf",width=6.5,height=5)
s.class(cstar=1,addaxes=TRUE, grid=TRUE, axesell=TRUE,
        dfxy=AFC$li, fac=as.factor(PourCAH$clusters), col=1:10,
        label=c(1:10), csub=1.2, possub="bottomright")
#dev.off()
```

```
#################################################
#### Barplots describing profiles of clusters ####
#################################################

clusProfile <- aggregate(PourCAH[, 1:12],  #Nb of variables
                         by = list(PourCAH$clusnames),
                         mean)
colnames(clusProfile)[1] <- "CLUSTER"
clusLong <- melt(clusProfile, id.vars = "CLUSTER")

# plot diagram
g <- ggplot(clusLong) +
  geom_bar(aes(x = variable, y = value, fill = CLUSTER),
           stat = "identity") + 
  facet_wrap(~ CLUSTER) + coord_flip() + 
  scale_fill_manual(values = cahPal)

g
#ggsave(g, file=sprintf("analyses_carroyage_1km_avec_stewart/typo_cah_resultats_R/diagramme_by_clus.pdf"))

# plot multiple lines. 
# cf. http://www.markhneedham.com/blog/2014/09/16/r-ggplot-plotting-multiple-variables-on-a-line-chart/
######################
#Create a year variable
clusLong$s <- as.character(clusLong$variable)  
clusLong$s1 <- as.numeric(gsub("[^0-9]","", clusLong$s))

g <- ggplot(clusLong, aes(x = s1, y = value, colour = CLUSTER)) + 
  geom_line() + 
  ylab(label="Potential Price (Eur)") + 
  xlab("Year") + 
  scale_colour_manual(values = cahPal) +
  scale_x_continuous(breaks = c(1996,1999,2003:2012))+
  scale_y_continuous(labels = scales::comma)
g
#ggsave(g, file=sprintf("analyses_carroyage_1km_avec_stewart/typo_cah_resultats_R/evol_by_clus.pdf"))

# z values / normalized plot
######################
rownames(clusProfile) <- as.character(clusProfile$CLUSTER) #GEOID
clusProfiled <- clusProfile
clusProfiled$CLUSTER <- NULL

clusProfileStd <- scale(clusProfiled)

clusLongStd <- melt(clusProfileStd, id.vars = "rownames")
clusLongStd$s <- as.character(clusLongStd$X2)  
clusLongStd$s1 <- as.numeric(gsub("[^0-9]","", clusLong$s))
clusLongStd$cluster <- as.factor(clusLongStd$X1)

g <- ggplot(clusLongStd, aes(x = s1, y = value, colour = cluster)) + 
  geom_line() + 
  ylab(label="Normalized Potential Price (Eur)") + 
  xlab("Year") + 
  scale_colour_manual(values = cahPal)+
  scale_x_continuous(breaks = c(1996,1999,2003:2012))
g
#ggsave(g, file=sprintf("analyses_carroyage_1km_avec_stewart/typo_cah_resultats_R/evol_by_clus_std.pdf"))


############################################
#### Mapping cluster analysis of prices ####
############################################

#Spatial join
PourCAH$GEOID <- row.names(PourCAH)
PourCAH$nb <- 1

# Create a DBF file to be used later for comparison with other results
#Write DBF
library(foreign)
write.dbf(PourCAH,'Profile_classes_prix_cah_10classes.dbf')

#WRITE TEMP R data
FINAL_CAH_PRIX <- PourCAH

str(centroidesCarroyage@data$ID)
centroidesCarroyage@data$GEOID <- as.character(centroidesCarroyage$ID)

# Draw the basemap region
#pdf(file=sprintf("analyses_carroyage_1km_avec_stewart/typo_cah_resultats_R/typo_evol_newcarto_FR_nolabel.pdf", currentYear), width=8, height=7,useDingbats=FALSE)
opar <- par(mar = c(0,0,1.1,0))
plot(CartoMask, add = F, border = "NA", bg = "#cdd2d4")
# Map the typology
# Map sans légende pour coller avec les graphiques...

propSymbolsTypoLayer(spdf = centroidesCarroyage, df = PourCAH,
                     spdfid = "GEOID", dfid = "GEOID",
                     var = "nb", var2="clusnames", col = cahPal, 
                     #                      k=0.00006, 
                     inches = 0.06, 
                     symbols = "square", lw= NULL, border = NULL,
                     legend.var.pos = "n",
                     legend.var2.pos = "n",
                     #                 border = "grey90", lwd = 0.2,
                     legend.var2.title.txt = "Traj. prix",
                     add=T)
plot(Fleuves, add=T, lwd = 1, border = "blue", col = "blue")
plot(departements_carto, add=T, lwd = 0.5, border = "black")

# Set a text to explicit the function parameters
#text(x = 582000, y = 6785000, 
#     labels = "Typology of single family homes price change between 1996 and 2012. \n Calculated on est. price of single family homes, \n Chi2 metric, Ward, r2=28%, grid of 7238 obs.", 
#     cex = .5, adj = 0, font = 1)
#Labels

labelLayer(spdf = labels, df = labels@data, txt = "NOM_COMM", 
           col = "black", cex = 0.4, font = 1)

#labelLayer(spdf = labels, df = labels@data, txt = "INSEE_COM", 
#           col = "black", cex = 0.4, font = 1)
layoutLayer(title = "Typology of house price dynamics, 1996-2012", 
            sources = "Source : Base BIEN Chambre des Notaires IDF", 
            author = "R. Le Goix, 2017, UMR Géographie-cités, Labex Dynamite")

par(opar)
#dev.off()
```

# 3. Using annual sellers-buyers balance to analyze neighborhood change

We use the information describing the sellers and buyers’ socio-occupational category : workers, intermediate occupation, salaried employees, executives, independent workers and retirees.

## Univariate overview of sellers and buyers

```
#####################################################################
############ PART 2 - SELLERS AND BUYERS ANALYSIS ##################
#####################################################################


##### Ces histo sont première approx et ne tiennent pas compte des effectifs pondérés.
g <- ggplot(dfdata) +
  geom_histogram (aes(x = annee),
                  stat = "bin", binwidth = 1) + 
  facet_wrap(~ CSP_AC) + 
  theme_bw() +
  xlab("year") +
  ggtitle("Count of buyers in actual transactions")

g
#ggsave(g, file=sprintf("analyses_carroyage_1km_avec_stewart/buyers_histo_all_short.pdf"))


g <- ggplot(dfdata) +
  geom_histogram (aes(x = annee),
                  stat = "bin", binwidth = 1) + 
  facet_wrap(~ CSP_VE) + 
  theme_bw() +
  xlab("year") +
  ggtitle("Count of sellers in actual transactions")

g

#ggsave(g, file=sprintf("analyses_carroyage_1km_avec_stewart/sellers_histo_al_shortl.pdf"))
```

## Sellers-buyers balance

Second, using sellers-buyers balance by categories as input variables describing each cell, a cluster analysis (ward method, euclidian distance) describes the categories for each neighborhood at each given date, assuming the different socio-occupational net balances are significant in analyzing local trends.

```
##############################################################
###### Loops to prepare profiles in line for each year  ######
##############################################################
# results of iterations are stored in carroyage

row.names(centroidesCarroyage) <- centroidesCarroyage$ID

#### Buyers ####

mesAnnees <- unique(dfdata$annee) 
mesAnnees <- sort(mesAnnees)
mesCSP <- c("2_IND","3_EXE","4_INT","5_EMP","6_WOR","7_RET","8_OTH")


#Uncomment to get shortcuts for testing purpose
#mesCSP <- c("3_EXE","4_INT")
#mesAnnees <- c(2012)
#currentCSP <- c("3_EXE")
#currentYear <- c(2012)


for (currentYear in mesAnnees) {
  for (currentCSP in mesCSP) {
    
    ### Determine code PCS for map#
    temp <- subset(CS_Cat_Fr_EN, CATSOC_CODE == currentCSP)
    currentCSPlong <- as.character(temp$CATSOC_LIB_EN)
    label_boucle <- print (paste(currentCSPlong, currentYear, sep = " - "))
    label_boucle_short <- print (paste(currentCSP, currentYear, sep = " - "))
 
   #Subset for current year and current CSP
    currentData <- dfdata[!is.na(dfdata$CSP_AC) &
                                         dfdata$CSP_AC == currentCSP &
                                         dfdata$annee == currentYear, ]
    
    
    #### Aggregate data into KNOWNPOINTS 
    Carreau_sum <- aggregate(currentData[, c("weight")],
                             by = list(currentData$id_carreau),
                             sum)
    
    ### Create knownpoints
    names(Carreau_sum)[1] <- "ID"
    names(Carreau_sum)[2] <- "weight"
    Carreau_sum$id <- as.character(Carreau_sum$ID)
    knownpoints <- merge(centroidesCarroyage, Carreau_sum, by = "ID")    
    knownpoints <- knownpoints[!is.na(knownpoints@data$weight) ,] #  Remove rows in spatial dataframe
    
    # Stewart interpolation
    currentStewartACQ <- stewart(knownpts = knownpoints,
                                 unknownpts = centroidesCarroyage,
                                 matdist = DistMatrix, varname = "weight",
                                 typefct = "pareto", span = 10, 
                                 beta = -0.27, longlat = FALSE)
    currentStewartACQ@data$nb <- 1 #Trick for mapping purpose
    # Si le calcul de bornesQuantiles intervient à chaque boucle
    bornesQuantiles <- quantile(currentStewartACQ@data$OUTPUT, # determine quantile for cartography
                                probs = seq(0,1, 0.2),
                                na.rm=TRUE)
    
    
    ##### MAP BUYER : optional. Uncomment to get the map output. time consuming
    #Blue for buyers
#     colPal <- carto.pal(pal1 = "blue.pal" ,n1 = 5)
#     # Draw the basemap region
# #    pdf(file=sprintf("analyses_carroyage_1km_avec_stewart/par_annee/_%s_BUYERS_labels.pdf", label_boucle), width=8, height=7,useDingbats=FALSE)
#     opar <- par(mar = c(0,0,1.1,0))
#     plot(CartoMask, add = F, border = "NA", bg = "#cdd2d4")
#     # Map the regional potential price 2008
#     propSymbolsChoroLayer(spdf = currentStewartACQ, df = currentStewartACQ@data,
#                           spdfid = "ID", dfid = "ID",
#                           var = "nb", var2="OUTPUT",
#                           breaks = bornesQuantiles, col = colPal, 
#                           #                      k=0.00006, 
#                           inches = 0.06, 
#                           symbols = "square", lw= NULL, border = NULL,
#                           legend.var.pos = "n",
#                           legend.var2.pos = "topright",
#                           #                 border = "grey90", lwd = 0.2,
#                           legend.var2.title.txt = "Density",
#                           legend.var2.values.rnd = 2,
#                           add=T)
# 
# 
#     plot(departements_carto, add=T, lwd = 0.5, border = "black")
#     # Set a text to explicit the function parameters
#     text(x = 590000, y = 6790000, 
#          labels = label_boucle, 
#          cex = 1.0, adj = 0, font = 2)
#     #Labes
#     labelLayer(spdf = labels, df = labels@data, txt = "NOM_COMM", 
#                col = "black", cex = 0.4, font = 1)
#     layoutLayer(title = "Density of buyers (Potential in a 10 min. radius)", 
#                 sources = "Source : Base BIEN Chambre des Notaires IDF", 
#                 author = "R. Le Goix, 2016, UMR Géographie-cités, Labex Dynamite")
#     
#     par(opar)
#     #dev.off()   
    
#### Sellers ####
    
    currentData <- dfdata[!is.na(dfdata$CSP_VE) &
                                         dfdata$CSP_VE == currentCSP &
                                         dfdata$annee == currentYear, ]
    
    Carreau_sum <- aggregate(currentData[, c("weight")],
                             by = list(currentData$id_carreau),
                             sum)
    
      ### Create knownpoints
      names(Carreau_sum)[1] <- "ID"
      names(Carreau_sum)[2] <- "weight"
      Carreau_sum$id <- as.character(Carreau_sum$ID)
      knownpoints <- merge(centroidesCarroyage, Carreau_sum, by = "ID")    
      knownpoints <- knownpoints[!is.na(knownpoints@data$weight) ,] #  Remove rows in spatial dataframe

    currentStewartVE <- stewart(knownpts = knownpoints,
                                unknownpts = centroidesCarroyage,
                                matdist = DistMatrix, varname = "weight",
                                typefct = "pareto", span = 10, 
                                beta = -0.27, longlat = FALSE)
    currentStewartVE@data$nb <- 1 #Var indispensa
    # Si le calcul de bornesQuantiles intervient à chaque boucle
    bornesQuantiles <- quantile(currentStewartVE@data$OUTPUT, #attention prix div par 1000
                                probs = seq(0,1, 0.2),
                                na.rm=TRUE)
    
    
    ##### MAP SELLERS #Optional
    # # Orange for sellers
    # colPal <- carto.pal(pal1 = "orange.pal" ,n1 = 5)
    # # Draw the basemap region
    # #pdf(file=sprintf("analyses_carroyage_1km_avec_stewart/par_annee/_%s_SELLERS_labels.pdf", label_boucle), width=8, height=7,useDingbats=FALSE)
    # opar <- par(mar = c(0,0,1.1,0))
    # plot(CartoMask, add = F, border = "NA", bg = "#cdd2d4")
    # # Map
    # propSymbolsChoroLayer(spdf = currentStewartVE, df = currentStewartVE@data,
    #                       spdfid = "ID", dfid = "ID",
    #                       var = "nb", var2="OUTPUT",
    #                       breaks = bornesQuantiles, col = colPal, 
    #                       inches = 0.06, 
    #                       symbols = "square", lw= NULL, border = NULL,
    #                       legend.var.pos = "n",
    #                       legend.var2.pos = "topright",
    #                       legend.var2.title.txt = "Density",
    #                       legend.var2.values.rnd = 2,
    #                       add=T)
    # plot(departements_carto, add=T, lwd = 0.5, border = "black")
    # # Set a text to explicit the function parameters
    # text(x = 590000, y = 6790000, 
    #      labels = label_boucle, 
    #      cex = 1.0, adj = 0, font = 2)
    # labelLayer(spdf = labels, df = labels@data, txt = "NOM_COMM", 
    #            col = "black", cex = 0.4, font = 1)
    # layoutLayer(title = "Density of sellers (Potential in a 10 min. radius)", 
    #             sources = "Source : Base BIEN Chambre des Notaires IDF", 
    #             author = "R. Le Goix, 2016, UMR Géographie-cités, Labex Dynamite")
    # 
    # par(opar)
    # #dev.off()   
    
    
    ###### SELLER BUEYR BALANCE ######
    ### Create temp currentStewart DF for alteration
    currentStewart <- carroyage
    currentStewart@data$current_solde_acq_ven <-   currentStewartACQ@data$OUTPUT - currentStewartVE@data$OUTPUT
    currentStewart@data$nb <- 1 #Var indispensa
    currentName <- paste("SOLDE", currentCSP, sep="")
    currentName
    summary(currentStewart@data$current_solde_acq_ven)
    bornesQuantiles <- quantile(currentStewart@data$current_solde_acq_ven, 
                                probs = seq(0,1, 0.2),
                                na.rm=TRUE)
    colPal <- rev(brewer.pal(n = 5, name = "RdYlBu"))
    
    # Draw the basemap region
    #pdf(file=sprintf("analyses_carroyage_1km_avec_stewart/par_annee/_%s_solde_labels.pdf", label_boucle), width=8, height=7,useDingbats=FALSE)
    opar <- par(mar = c(0,0,1.1,0))
    plot(CartoMask, add = F, border = "NA", bg = "#cdd2d4")
    # Map the regional potential price 2008
    propSymbolsChoroLayer(spdf = currentStewart, df = currentStewart@data,
                          spdfid = "ID", dfid = "ID",
                          var = "nb", var2="current_solde_acq_ven",
                          breaks = bornesQuantiles, col = colPal, 
                          #                      k=0.00006, 
                          inches = 0.06, 
                          symbols = "square", lw= NULL, border = NULL,
                          legend.var.pos = "n",
                          legend.var2.pos = "topright",
                          #                 border = "grey90", lwd = 0.2,
                          legend.var2.title.txt = "Balance",
                          legend.var2.values.rnd = 2,
                          add=T)
    plot(departements_carto, add=T, lwd = 0.5, border = "black")
    # Set a text to explicit the function parameters
    text(x = 590000, y = 6790000, 
         labels = label_boucle, 
         cex = 1.0, adj = 0, font = 2)
    #Labels
    labelLayer(spdf = labels, df = labels@data, txt = "NOM_COMM", 
               col = "black", cex = 0.4, font = 1)
    layoutLayer(title = "Balance of buyers - sellers (Potential in a 10 min. radius)", 
                sources = "Source : Base BIEN Chambre des Notaires IDF", 
                author = "R. Le Goix, 2016, UMR Géographie-cités, Labex Dynamite")
    
    par(opar)
    #dev.off()   
    
    #Compilation des résultats     #ici prix non arrondis et valeur entière
    current_solde_acq_ven <-   currentStewartACQ@data$OUTPUT - currentStewartVE@data$OUTPUT
    currentName <- paste("SOLDE", currentCSP, sep="")
    carroyage[[currentName]] <- current_solde_acq_ven
    currentName <- paste("ACQ", currentCSP, sep="")
    carroyage[[currentName]] <- currentStewartACQ@data$OUTPUT
    currentName <- paste("VE", currentCSP, sep="")
    carroyage[[currentName]] <- currentStewartVE@data$OUTPUT
    
    rm(currentData, currentMatrix, currentStewartACQ, currentStewartVE, currentStewart)
    
    
    
  }
  

  carroyage2 <- carroyage@data
#  write.dbf(carroyage2, file=sprintf("analyses_carroyage_1km_avec_stewart/par_annee/_%s_carroyagesellersbuyers.dbf", currentYear))
  assign(paste("carroyage_acq_ven",currentYear,sep="_"), carroyage2)

}
```

### Correlation plots between sellers and buyers

This chunk of code prepares outpout for Pearson correlation matrices (heatmaps) for percent sellers and buyers in 1996, 2003 and 2012, in *S2 Fig* supporting information

```
####### 4.4 HEAT MAP CORRELATION MATRIX SELLERS BUYERS  #######

# Example for year 2003. Change source file carroyage_acq_ven_2003 in following lines.

totoacq <- carroyage_acq_ven_2003[,c("ACQ2_IND","ACQ3_EXE","ACQ4_INT","ACQ5_EMP","ACQ6_WOR","ACQ7_RET","ACQ8_OTH")]

totosell <- carroyage_acq_ven_2003[,c("VE2_IND","VE3_EXE","VE4_INT","VE5_EMP","VE6_WOR","VE7_RET","VE8_OTH")]

### FUN Calc pct ACQ
total_col = apply(totoacq, 1, sum)
pcts = lapply(totoacq, function(x) {
  x / total_col
})
### pcts as df
pctsacq = as.data.frame(pcts)

### FUN Calc pct VE
total_col = apply(totosell, 1, sum)
pcts = lapply(totosell, function(x) {
  x / total_col
})
### pcts as df
pctsell = as.data.frame(pcts)


##### Correlogram / heat maps
toto <- cbind(pctsacq,pctsell)
toto<-toto[complete.cases(toto),]

#corr <- round(cor(toto), 2)

# Correlation matrix from df
# with VE as rows 
# and ACQ columns 
x <- toto[1:7]
y <- toto[8:14]
corr <- round(cor(x, y), 2)


df <- reshape2::melt(corr)
gg <- ggplot(df, aes(x=Var1, y=Var2, fill=value, label=value)) + geom_tile() + theme_bw() + geom_text(aes(label=value, size=value), color="white") + labs(title="2003 - Correlation plot",x ="Buyers", y = "Sellers") + theme(text=element_text(size=10), legend.position="none")

gg

p3 <- gg + scale_fill_gradient2()
p3
```

## Sellers-buyers typology

Because of the large number of permutations (94956 rows and 7 variables), we opted for the `fastcluster` library that implements a more efficient though stable algorithm (Müllner 2013).

This chunk of code generates the file of sellers-buyers balance, made available as *Supporting information S6 File*.

```
########################
####### Typology #######
########################


#### Concatenate annual files and keep only seller-buyers balance values

toto1996 <- carroyage_acq_ven_1996[, c("ID","SOLDE2_IND","SOLDE3_EXE","SOLDE4_INT","SOLDE5_EMP","SOLDE6_WOR","SOLDE7_RET","SOLDE8_OTH")]
toto1999 <- carroyage_acq_ven_1999[, c("ID","SOLDE2_IND","SOLDE3_EXE","SOLDE4_INT","SOLDE5_EMP","SOLDE6_WOR","SOLDE7_RET","SOLDE8_OTH")]
toto2003 <- carroyage_acq_ven_2003[, c("ID","SOLDE2_IND","SOLDE3_EXE","SOLDE4_INT","SOLDE5_EMP","SOLDE6_WOR","SOLDE7_RET","SOLDE8_OTH")]
toto2004 <- carroyage_acq_ven_2004[, c("ID","SOLDE2_IND","SOLDE3_EXE","SOLDE4_INT","SOLDE5_EMP","SOLDE6_WOR","SOLDE7_RET","SOLDE8_OTH")]
toto2005 <- carroyage_acq_ven_2005[, c("ID","SOLDE2_IND","SOLDE3_EXE","SOLDE4_INT","SOLDE5_EMP","SOLDE6_WOR","SOLDE7_RET","SOLDE8_OTH")]
toto2006 <- carroyage_acq_ven_2006[, c("ID","SOLDE2_IND","SOLDE3_EXE","SOLDE4_INT","SOLDE5_EMP","SOLDE6_WOR","SOLDE7_RET","SOLDE8_OTH")]
toto2007 <- carroyage_acq_ven_2007[, c("ID","SOLDE2_IND","SOLDE3_EXE","SOLDE4_INT","SOLDE5_EMP","SOLDE6_WOR","SOLDE7_RET","SOLDE8_OTH")]
toto2008 <- carroyage_acq_ven_2008[, c("ID","SOLDE2_IND","SOLDE3_EXE","SOLDE4_INT","SOLDE5_EMP","SOLDE6_WOR","SOLDE7_RET","SOLDE8_OTH")]
toto2009 <- carroyage_acq_ven_2009[, c("ID","SOLDE2_IND","SOLDE3_EXE","SOLDE4_INT","SOLDE5_EMP","SOLDE6_WOR","SOLDE7_RET","SOLDE8_OTH")]
toto2010 <- carroyage_acq_ven_2010[, c("ID","SOLDE2_IND","SOLDE3_EXE","SOLDE4_INT","SOLDE5_EMP","SOLDE6_WOR","SOLDE7_RET","SOLDE8_OTH")]
toto2011 <- carroyage_acq_ven_2011[, c("ID","SOLDE2_IND","SOLDE3_EXE","SOLDE4_INT","SOLDE5_EMP","SOLDE6_WOR","SOLDE7_RET","SOLDE8_OTH")]
toto2012 <- carroyage_acq_ven_2012[, c("ID","SOLDE2_IND","SOLDE3_EXE","SOLDE4_INT","SOLDE5_EMP","SOLDE6_WOR","SOLDE7_RET","SOLDE8_OTH")]

toto1996$annee <- 1996
toto1999$annee <- 1999
toto2003$annee <- 2003
toto2004$annee <- 2004
toto2005$annee <- 2005
toto2006$annee <- 2006
toto2007$annee <- 2007
toto2008$annee <- 2008
toto2009$annee <- 2009
toto2010$annee <- 2010
toto2011$annee <- 2011
toto2012$annee <- 2012

PourTypo <- rbind (toto1996,toto1999,toto2003,toto2004,toto2005,toto2006,toto2007,toto2008,toto2009,toto2010,toto2011,toto2012)
rm(toto1996,toto1999,toto2003,toto2004,toto2005,toto2006,toto2007,toto2008,toto2009,toto2010,toto2011,toto2012)
#pourtypo 465960 obs and 9 variables

mesAnnees <- unique(PourTypo$annee) 
mesAnnees <- sort(mesAnnees)

### Create a new row.id with year and GEOID : each spatial entity (cell) is described for each given year, in line.
PourTypo$idYear <- paste(PourTypo$annee,"&",PourTypo$ID,sep="")
PourTypo$annee<-NULL
PourTypo$ID<-NULL

head(PourTypo)
write.csv(PourTypo,"S6_seller_buyer_balance_1kgrid_1996_2012.csv")
```

### Cluster analysis of sellers-buyers balance, average profiles and z-value plots, map

```
###################################
######  Cluster analysis ##########
###################################

# To replicate, Load Supporting information S6 File : S6_seller_buyer_balance_1kgrid_1996_2012.csv 
# and merge it within 'carroyage@data' spatial data frame
#PourTypo <- read.csv("S6_seller_buyer_balance_1kgrid_1996_2012.csv")

#Normalize data
PourTypo.Temp <- PourTypo
PourTypo.Temp$idYear <- NULL

PourTypo.norm <- scale(PourTypo.Temp)
rm(PourTypo.Temp)

#Color palette for final typology
cahPal <- c( "#ffff99", "#b2df8a", "#33a02c", "#a6cee3", "#fdbf6f","#ff7f00" , "#b15928", "#e31a1c","#1f78b4", "#6a3d9a","#cab2d6") 

#pie(rep(1, 11), col = cahPal) #Checking palette.

#Load fastcluster library
library(fastcluster)

HClust.1 <- hclust.vector(PourTypo.norm, method = "ward", members = NULL, metric = "euclidean",
                          p = NULL)

HClust.1$height <- HClust.1$height^2

plot(HClust.1, main= "Clusters dendrogram", xlab= 
       "n=94956", 
     sub="", labels=FALSE)
rect.hclust(HClust.1, k=11, border=cahPal)

sortedHeight <- sort(HClust.1$height, decreasing = TRUE) 

head(sortedHeight, 13L)

#pdf(file="analyses_carroyage_1km_avec_stewart/typo_cah_trajectoires_v3/synthese_finales.pdf",width=6.5,height=5)
par(mfrow=c(2,3))

dend <- as.dendrogram(HClust.1)
plot(cut(dend, h = 12510)$upper, 
     main="Upper tree of cut at k=11 classes")
rect.hclust(HClust.1, k=11, border=cahPal) # On retient 11 classes, meilleur compromis

plot(sortedHeight,
     type = "h",
     xlab = "Node",
     ylab = "Aggregation level")

relHeight <- sortedHeight / sum(sortedHeight) *100
cumHeight <- cumsum(relHeight)
barplot(relHeight[1:30], names.arg = seq(1, 30, 1),
        col = "black", border = "white", xlab = "Node", ylab = "Share of total inertia (%)")

barplot(relHeight[5:20], names.arg = seq(5, 20, 1),
        col = "black", border = "white", xlab = "Node", ylab = "Share of total inertia (%)")

barplot(cumHeight[1:30], names.arg = seq(1, 30, 1), col = "black", border = "white",
        xlab = "Nb of clusters",
        ylab = "Share of total inertia (%)")

barplot(cumHeight[5:20], names.arg = seq(5, 20, 1), col = "black", border = "white",
        xlab = "Nb of clusters",
        ylab = "Share of total inertia (%)")

#dev.off()


clusters <- cutree(HClust.1, k = 11) # 11 clusters solution
# append cluster assignment for absolute values
PourTypo <- data.frame(PourTypo, clusters)

# append cluster assignment for relative values
PourTypo.norm <- data.frame(PourTypo.norm, clusters)


##########################################
######  Absolute values diagram ##########
##########################################
rm(cluster_means)
cluster_means <- aggregate(PourTypo,by=list(clusters),FUN=mean)


colnames(cluster_means)[1] <- "CLUSTER"
cluster_means$clusters <- NULL
cluster_means$idYear <- NULL
clusLong <- melt(cluster_means, id.vars = "CLUSTER")
clusLong$clusters <- as.factor(clusLong$CLUSTER)

# plot diagram
g <- ggplot(clusLong) +
  geom_bar(aes(x = variable, y = value, fill = clusters),
           stat = "identity") + 
  facet_wrap(~ CLUSTER) + coord_flip() + 
  scale_fill_manual(values = cahPal) +
  theme(text = element_text(size=12),
        axis.text.y = element_text(size=6 ))

g
#ggsave(g, file=sprintf("analyses_carroyage_1km_avec_stewart/typo_cah_trajectoires_v3/diagram_by_clus_zscores.pdf"), width=28, height=20, units=c("cm"))


################################
###### Z-values diagram ########
################################
rm(clusLong.norm)
cluster_means.norm <- aggregate(PourTypo.norm,by=list(clusters),FUN=mean)
cluster_means.norm$idYear <- NULL

colnames(cluster_means.norm)[1] <- "CLUSTER"
cluster_means.norm$clusters <- NULL
#cluster_means.norm$fit.cluster.1 <- NULL
clusLong.norm <- melt(cluster_means.norm, id.vars = "CLUSTER")
clusLong.norm$clusters <- as.factor(clusLong.norm$CLUSTER)


# plot diagram
g <- ggplot(clusLong.norm) +
  geom_bar(aes(x = variable, y = value, fill = clusters),
           stat = "identity") + 
  facet_wrap(~ CLUSTER) + coord_flip() + 
  scale_fill_manual(values = cahPal) +
  theme(text = element_text(size=12),
        axis.text.y = element_text(size=6 ))

g
#ggsave(g, file=sprintf("analyses_carroyage_1km_avec_stewart/typo_cah_trajectoires_v3/diagram_by_clus_zscores_relative_values.pdf"), width=28, height=20, units=c("cm"))


##########################################
########## Map final results #############
##########################################

##### Split GEOID and year
PourTypo$date <- sapply(strsplit(PourTypo$idYear, split='&', fixed=TRUE), function(x) (x[1]))
PourTypo$id <- sapply(strsplit(PourTypo$idYear, split='&', fixed=TRUE), function(x) (x[2]))
PourTypo$nb <- 1 #Indispensable pour carto

##### Define Loop
for (currentYear in mesAnnees) {
  
  toto<-subset(PourTypo, date==currentYear)
  # Draw the basemap region
#  pdf(file=sprintf("analyses_carroyage_1km_avec_stewart/typo_cah_trajectoires_v3/_%s_typoCAH_traj.pdf", currentYear), width=8, height=7,useDingbats=FALSE)
  opar <- par(mar = c(0,0,1.1,0))
  #opar <- par(mar = c(0,0,0,0))
  plot(CartoMask, add = F, border = "NA", bg = "#cdd2d4")
  # Map the regional potential p
  propSymbolsTypoLayer(spdf = carroyage, df = toto,
                       spdfid = "ID", dfid = "id",
                       var = "nb", var2="clusters", col = cahPal, 
                        inches = 0.06, 
                       symbols = "square", lw= NULL, border = NULL,
                       legend.var.pos = "n",
                        legend.var2.pos = "n",
                       legend.var2.title.txt = "Clusters",
                       add=T)
  
  plot(departements_carto, add=T, lwd = 0.5, border = "black")
  text(x = 590000, y = 6790000, 
       labels = currentYear, 
       cex = 2, adj = 0, font = 2)
  #Labels
  labelLayer(spdf = labels, df = labels@data, txt = "NOM_COMM", 
             col = "black", cex = 0.4, font = 1)
  layoutLayer(title = "Typology of sellers - buyers balance - 1 km grid", 
             sources = "Source : Base BIEN Chambre des Notaires IDF", 
             author = "R. Le Goix, 2018, UMR Géographie-cités, Labex Dynamite")
  
  par(opar)
#  dev.off()   
  
}
```

# 4. Longitudinal categorical sequences of neighborhoods

Given the aforementioned sellers-buyers typology, described for each given year, the last series of results derive from the sequencing of consecutive states for each neighborhood. The R `TraMiner` package algorithms were designed to sort out and describe in sequences the successive states of neighborhoods: this allows us to analyze local change as sequences and permutations between different states.

```
####################################
###### Traminer Analysis ###########
####################################
library(TraMineR)
library('reshape2')

##### 4.1 Put results together ####
toto <- PourTypo[c("id","date","clusters")]

clusters_by_year <- dcast(toto, id ~ date)

##### Text for nterpretation of results
mvad.alphabet <- c("1", "2", "3", "4", "5", "6", "7", "8","9","10","11")
mvad.labels <- c("1. Stable / maturing exurbs, villages and rural areas", 
                 "2. Stable exurban markets", 
                 "3. Average suburban profile (Interm Occ. & Exec)", 
                 "4. Upper segments in the inner suburbs", 
                 "5. Mature stable middle-class suburbs", 
                 "6. Dynamic mixed inner suburbs markets", 
                 "7. Middle class / intermediary market", 
                 "8. Workers refuges",
                 "9. Golden suburban ghettos",
                 "10. Very dynamic mixed markets",
                 "11. Very dynamic upper-middle markets in mixed mature suburbs"
                 )
mvad.scodes <- c("StabRuralOuterSubs", "StabExurbMarkets", "AvProfile", "Upper segments",
                 "MatStabMidClSub", "DynMixedInnerSub", "Intermedmarkets", "WorkersRef",
                 "GoldGhetto","DynMixedMarkets", "DynUpperMiddleClass")


mvad.seq <- seqdef(clusters_by_year, 2:13, cpal = cahPal, alphabet = mvad.alphabet, states = mvad.scodes, 
                   labels = mvad.labels, xtstep = 1)
summary(mvad.seq)

##### Analyze first sequences ####
seqstatd(mvad.seq)

#pdf(file= paste("analyses_carroyage_1km_avec_stewart/TraMineR/sequence_analysis.pdf"), width = 11.7, height = 8.27)
par(mfrow = c(2, 2))
seqiplot(mvad.seq, title = "a. Index plot (first 10 sequences)", withlegend = FALSE, border = NA)
seqIplot(mvad.seq, title = "b. State distribution plot", sortv = "from.start", withlegend = FALSE)
seqfplot(mvad.seq, title = "c. 10 most frequent sequence", withlegend = FALSE, border = NA)
seqlegend(mvad.seq)
#dev.off()

#pdf(file= paste("analyses_carroyage_1km_avec_stewart/TraMineR/descriptive_statistics.pdf"), width = 11.7, height = 8.27)
par(mfrow = c(2, 2))
seqdplot(mvad.seq, title = "a. State distribution (Freq)", withlegend = FALSE, border = NA)
seqHtplot(mvad.seq, title = "b. Entropy index")
seqmsplot(mvad.seq,  title = "c. Mean time", withlegend = FALSE, border = NA)
seqmtplot(mvad.seq, title = "d. Modal state seq.", withlegend = FALSE)
#dev.off()

#pdf(file= paste("analyses_carroyage_1km_avec_stewart/TraMineR/traminer_sequences_edition.pdf"), width = 11.7, height = 8.27)
seqIplot(mvad.seq, title = "Sequence plot", sortv = "from.start", withlegend = FALSE)
#dev.off()

#pdf(file= paste("analyses_carroyage_1km_avec_stewart/TraMineR/traminer_etats_edition.pdf"), width = 11.7, height = 8.27)
seqdplot(mvad.seq, title = "State distribution (Freq)", withlegend = T, border = NA)
#dev.off()


# Prepare typology
dist.om1 <- seqdist(mvad.seq, method = "OM", indel = 1, sm = "TRATE", with.missing=TRUE)
library(cluster)
clusterward1 <- agnes(dist.om1, diss = TRUE, method = "ward")

clusterward1$height2 <- clusterward1$height^2
sortedHeight <- sort(clusterward1$height2, decreasing = TRUE) 
head(sortedHeight, 20)

#pdf(file="analyses_carroyage_1km_avec_stewart/TraMineR/TypoFinaleTraj_synthese.pdf",width=6.5,height=5)
par(mfrow=c(2,2))


plot(sortedHeight,
     type = "h",
     xlab = "Noeuds",
     ylab = "Niveau d'agrégation")
relHeight <- sortedHeight / sum(sortedHeight) * 100 
cumHeight <- cumsum(relHeight)

head(cumHeight, 20)
barplot(relHeight[1:30], names.arg = seq(1, 30, 1),
        col = "black", border = "white", xlab = "Noeuds", ylab = "Part de l'inertie totale (%)")

barplot(cumHeight[1:30], names.arg = seq(1, 30, 1), col = "black", border = "white",
        xlab = "Nombre de classes",
        ylab = "Part de l'inertie totale (%)")

#plot tree
plot(clusterward1, which.plot = 2, labels=FALSE)
rect.hclust(clusterward1, k=6, border="red") #6 classes et 70% variance estimée

#dev.off()

cl1.9 <- cutree(clusterward1, k = 6)
cl1.9fac <- factor(cl1.9, labels = paste("Type", 1:6))


##### plot typology ####

#pdf(file= paste("analyses_carroyage_1km_avec_stewart/TraMineR/typo_sequence.pdf"), width = 8.27, height = 11.7)
seqIplot(mvad.seq, group = cl1.9fac, sortv = "from.start")
#dev.off()


#pdf(file= paste("analyses_carroyage_1km_avec_stewart/TraMineR/typo_state_sequence.pdf"), width = 8.27, height = 11.7)
seqdplot(mvad.seq, group = cl1.9fac, border = NA)
#dev.off()

#pdf(file= paste("analyses_carroyage_1km_avec_stewart/TraMineR/plot_representative_seq.pdf"), width = 10, height = 15)
seqrplot(mvad.seq, dist.matrix = dist.om1, group = cl1.9fac,
         border = NA)
#dev.off()
seqlegend(mvad.seq)

cpal(mvad.seq)
```

# 5. Correlation between dynamic of price and neighborhood trajectories

## Create line plot to analyse the dynamics of prices for each cluster of neighhborhood sequences

```
### df FINAL_CAH_PRIX countains description for price growth trajectories
### df cl1.9 countains description for clusters in traminer
### Create a clean file Traminer with typo description

# N=7888
# Merge both typos

Typo_prix_traminer <-data.frame(clusters_by_year,cl1.9)
Typo_prix_traminer$cl1.9 <-as.factor(cl1.9)

### Clean up files
Typo_prix_traminerTrimed <-  Typo_prix_traminer[c("id","cl1.9")]
colnames(Typo_prix_traminerTrimed)[2] <- "SellerBuyerTrajectory"
FINAL_CAH_PRIX$id <- FINAL_CAH_PRIX$GEOID

### Trim and merge BY ID both typologies
ForTrendComparisons <-  merge(FINAL_CAH_PRIX, Typo_prix_traminerTrimed , by = "id")


### ggplot line graphs by type
#################################################
#### Barplots describing profiles of clusters ####
#################################################

clusProfile_typo_compared <- aggregate(ForTrendComparisons[, 2:13],  #Nb of variables
                         by = list(ForTrendComparisons$SellerBuyerTrajectory),
                         mean)
colnames(clusProfile_typo_compared)[1] <- "CLUSTER"
clusLong <- melt(clusProfile_typo_compared, id.vars = "CLUSTER")


# plot multiple lines. 
# cf. http://www.markhneedham.com/blog/2014/09/16/r-ggplot-plotting-multiple-variables-on-a-line-chart/
######################
#Create a year variable
clusLong$s <- as.character(clusLong$variable)  
clusLong$s1 <- as.numeric(gsub("[^0-9]","", clusLong$s))

g <- ggplot(clusLong, aes(x = s1, y = value, colour = CLUSTER)) + 
  geom_line() + 
  ylab(label="Potential Price (Eur)") + 
  xlab("Year") + 
  scale_colour_manual(values = cahPal) +
  scale_x_continuous(breaks = c(1996,1999,2003:2012))+
  scale_y_continuous(labels = scales::comma)
g

#  normalized plot
######################
rownames(clusProfile_typo_compared) <- as.character(clusProfile_typo_compared$CLUSTER) #GEOID
clusProfile_typo_compared_d <- clusProfile_typo_compared
clusProfile_typo_compared_d$CLUSTER <- NULL

clusProfileStd <- scale(clusProfile_typo_compared_d)

clusLongStd <- melt(clusProfileStd, id.vars = "rownames")
clusLongStd$s <- as.character(clusLongStd$Var2)  
clusLongStd$s1 <- as.numeric(gsub("[^0-9]","", clusLong$s))
clusLongStd$cluster <- as.factor(clusLongStd$Var1)

g <- ggplot(clusLongStd, aes(x = s1, y = value, colour = cluster)) + 
  geom_line() + 
  ylab(label="Normalized Potential Price (Eur)") + 
  xlab("Year") + 
  scale_colour_manual(values = cahPal)+
  scale_x_continuous(breaks = c(1996,1999,2003:2012))
g
```

## Run a ChiSq test to analyze the correlation between

```
#######################################################################################################
######## Mosaic plot and chi2 test between typology of valeues  and neighborhood sequences #########
#######################################################################################################

### df FINAL_CAH_PRIX countains description for price growth trajectories
### df cl1.9 countains description for clusters in traminer
### Create a clean file Traminer with typo description

# N=7888
# Merge both typos

Typo_prix_traminer <-data.frame(clusters_by_year,cl1.9)
Typo_prix_traminer$cl1.9 <-as.factor(cl1.9)
Typo_prix_traminer$nb <- 1

### Clean up files
Typo_prix_traminerTrimed <-  Typo_prix_traminer[c("id","cl1.9")]
FINAL_CAH_PRIX_Trimed <-  FINAL_CAH_PRIX[c("GEOID","clusnames")]

colnames(FINAL_CAH_PRIX_Trimed)[1] <- "id"
colnames(FINAL_CAH_PRIX_Trimed)[2] <- "ValueTrajectory"
colnames(Typo_prix_traminerTrimed)[2] <- "SellerBuyerTrajectory"

### Trim and merge BY ID both typologies
ForMosaic <-  merge(FINAL_CAH_PRIX_Trimed, Typo_prix_traminerTrimed , by = "id")

#N:7238

#Recode for lisibility

# ForMosaic$ValueTrajectory <- Recode(ForMosaic$ValueTrajectory, 
#                           '"3"="Upper (3)"; "4"="Upper volatile after 2008 (4)"; "8"="Intermediate + stable (8)"; "2"="Intermediate - stable (2)"; "6"="Upper - volatile after 2004 (6)" ; "5"="Average - light depreciation (5)"; "7"="Lower depreciate 99 & after 2007 (7)"; "9"="Lower & volatile 2007-11 (9)"; "1"="Lower depreciate after 2006 (1)" ; "10"="Lower depreciate after 2009 (10)"')
# 
# ForMosaic$SellerBuyerTrajectory <- Recode(ForMosaic$SellerBuyerTrajectory, 
#                                     '"1"="Stable \'burbs (1)"; "2"="Stable but Retired sell - Interm/Exec buy (2)"; "3"="Transition to av. prof (3)"; "4"="To Interm dynamic markets (4)"; "5"="Volat. workers & interm. markets (5)"; "6"="Enduring golden suburban ghettos (6)"')

#With shorter names
ForMosaic$ValueTrajectory <- Recode(ForMosaic$ValueTrajectory, 
                                    '"3"="3/Upper"; "4"="4/UpVol08"; "8"="8/Interm+"; "2"="2/Interm-"; "6"="6/UpVol04" ; "5"="5/AverDeprec"; "7"="7/LowDeprec"; "9"="9/LowVol"; "1"="1/LowDeprec06" ; "10"="10/LowDeprec09"')

ForMosaic$SellerBuyerTrajectory <- Recode(ForMosaic$SellerBuyerTrajectory, 
                                          '"1"="1/Stable"; "2"="2/--Ret&+IntermExec"; "3"="3/Average"; "4"="4/IntermDynaMrkts"; "5"="5/Volat_workrs&interme"; "6"="6/GoldenGhettos"')


#### Mosaic
library(vcd)
library(car)

.Table <- xtabs(~ValueTrajectory+SellerBuyerTrajectory, data=ForMosaic)
.Table
.Test <- chisq.test(.Table, correct=TRUE)
.Test
.Test$expected # Expected Counts
round(.Test$residuals^2, 2) # Chi-square Components
#remove(.Test)
#remove(.Table)

#   Pearson's Chi-squared test

#data:  .Table
#X-squared = 2946.8, df = 45, p-value < 2.2e-16


#pdf(file= paste("analyses_carroyage_1km_avec_stewart/Mosaic/assoc_typoroad_traminer.pdf"), width = 11.7, height = 8.27)
assoc(.Table, shade=TRUE, legend=TRUE, 
       labeling_args=list(rot_labels=c(left=0,top=90),gp_labels=(gpar(fontsize=8)), 
                          pos_labels="center", just_labels = "right"), offset_labels = c(top = 4.2), offset_varnames = c(left = 10, top=5), margins = c(top = 6, left=5, right=5, bottom = 5))
#dev.off()
#pdf(file= paste("analyses_carroyage_1km_avec_stewart/Mosaic/mosaic_typoroad_traminer.pdf"), width = 11.7, height = 8.27)
mosaic(.Table, shade=TRUE, legend=TRUE,
       labeling_args=list(rot_labels=c(left=0,top=45),gp_labels=(gpar(fontsize=9)), 
                          pos_labels="center", just_labels = c(left="right",top="left")), offset_labels = c(top = 0), offset_varnames = c(left = 10, top=8), margins = c(top = 9, left=6, right=5, bottom = 0))
#dev.off()
```

# Session info

```
sessionInfo()
```

```
## R version 3.5.1 (2018-07-02)
## Platform: x86_64-apple-darwin15.6.0 (64-bit)
## Running under: macOS  10.14.2
## 
## Matrix products: default
## BLAS: /Library/Frameworks/R.framework/Versions/3.5/Resources/lib/libRblas.0.dylib
## LAPACK: /Library/Frameworks/R.framework/Versions/3.5/Resources/lib/libRlapack.dylib
## 
## locale:
## [1] en_US.UTF-8/en_US.UTF-8/en_US.UTF-8/C/en_US.UTF-8/en_US.UTF-8
## 
## attached base packages:
## [1] stats     graphics  grDevices utils     datasets  methods   base     
## 
## loaded via a namespace (and not attached):
##  [1] compiler_3.5.1  backports_1.1.2 magrittr_1.5    rprojroot_1.3-2
##  [5] tools_3.5.1     htmltools_0.3.6 yaml_2.2.0      Rcpp_0.12.19   
##  [9] stringi_1.2.4   rmarkdown_1.10  knitr_1.20      stringr_1.3.1  
## [13] digest_0.6.18   evaluate_0.12
```

# References

Charmes, E. 2009. “On the Residential ‘Clubbisation’ of French Periurban Municipalities.” *Urban Studies* 46 (1): 189–212. doi:10.1177/0042098008098642.

Commenges, H., and T. Giraud. 2016. “Introduction to the SpatialPosition package.” Cran. https://cran.r-project.org/web/packages/SpatialPosition/vignettes/SpatialPosition.html.

Commenges, H., T. Giraud, and J. Boulier. 2015. “SpatialPosition.” CRAN. https://cran.r-project.org/web/packages/SpatialPosition/index.html.

Grasland, C. 2009. “Spatial analysis of social facts. A tentative theoretical framework derived from tobler’s first law of geography and blau’s multilevel structural theory of society.” In *Handbook of Quantitative Geography*, edited by F. Bavaud and C. Mager, 46 p. University of Lausanne. http://halshs.archives-ouvertes.fr/halshs-00410669/.

Müllner, D. 2013. “fastcluster: Fast Hierarchical, Agglomerative Clustering Routines for R and Python.” *Journal of Statistical Software* 53 (9): 18. http://www.jstatsoft.org/v53/i09.

Stewart, J. Q. 1942. “A Measure of the Influence of a Population at a Distance.” *Sociometry* 5 (1): 63–71. doi:10.2307/2784954.

Stouffer, S. A. 1940. “Intervening opportunities : a theory relating mobility and distance.” *American Sociological Review* 5 (6): 845–67.

Tobler, W., and S. Wineburg. 1971. “A Cappadocian Speculation.” *Nature* 231 (5297): 39–41. doi:10.1038/231039a0.

Xiao, Y., C. Webster, and S. Orford. 2016. “Can street segments indexed for accessibility form the basis for housing submarket delineation?” *Housing Studies*, 1–23. doi:10.1080/02673037.2016.1150433.
